# Cost-effectiveness analysis of treatment with non-curative or palliative intent for hepatocellular carcinoma in the real-world setting

**DOI:** 10.1371/journal.pone.0185198

**Published:** 2017-10-10

**Authors:** Hla-Hla Thein, Yao Qiao, Ahmad Zaheen, Nathaniel Jembere, Gonzalo Sapisochin, Kelvin K. W. Chan, Eric M. Yoshida, Craig C. Earle

**Affiliations:** 1 Dalla Lana School of Public Health, University of Toronto, Toronto, Ontario, Canada; 2 Institute for Clinical Evaluative Sciences, Toronto, Ontario, Canada; 3 Department of Medicine, University of Toronto, Toronto, Ontario, Canada; 4 Multi-Organ Transplant, Division of General Surgery, University Health Network, University of Toronto, Toronto, Canada; 5 Odette Cancer Centre, Sunnybrook Health Sciences Centre, Toronto, Ontario, Canada; 6 Canadian Centre for Applied Research in Cancer Control (ARCC), Toronto, Ontario, Canada; 7 University of British Columbia, Division of Gastroenterology, Vancouver, BC, Canada; 8 Ontario Institute for Cancer Research, Toronto, Ontario, Canada; 9 Cancer Care Ontario, Toronto, Ontario, Canada; Chang Gung Memorial Hospital Kaohsiung Branch, TAIWAN

## Abstract

Hepatocellular carcinoma (HCC) presentation is heterogeneous necessitating a variety of therapeutic interventions with varying efficacies and associated prognoses. Poor prognostic patients often undergo non-curative palliative interventions including transarterial chemoembolization (TACE), sorafenib, chemotherapy, or purely supportive care. The decision to pursue one of many palliative interventions for HCC is complex and an economic evaluation comparing these interventions has not been done. This study evaluates the cost-effectiveness of non-curative palliative treatment strategies such as TACE alone or TACE+sorafenib, sorafenib alone, and non-sorafenib chemotherapy compared with no treatment or best supportive care (BSC) among patients diagnosed with HCC between 2007 and 2010 in a Canadian setting. Using person-level data, we estimated effectiveness in life years and quality-adjusted life years (QALYs) along with total health care costs (2013 US dollars) from the health care payer’s perspective (3% annual discount). A net benefit regression approach accounting for baseline covariates with propensity score adjustment was used to calculate incremental net benefit to generate incremental cost-effectiveness ratio (ICER) and uncertainty measures. Among 1,172 identified patients diagnosed with HCC, 4.5%, 7.9%, and 5.6%, received TACE alone or TACE+sorafenib, sorafenib, and non-sorafenib chemotherapy clone, respectively. Compared with no treatment or BSC (81.9%), ICER estimates for TACE alone or TACE+sorafenib was $6,665/QALY (additional QALY: 0.47, additional cost: $3,120; 95% CI: -$18,800-$34,500/QALY). The cost-effectiveness acceptability curve demonstrated that if the relevant threshold was $50,000/QALY, TACE alone or TACE+sorafenib, non-sorafenib chemotherapy, and sorafenib alone, would have a cost-effectiveness probability of 99.7%, 46.6%, and 5.5%, respectively. Covariates associated with the incremental net benefit of treatments are age, sex, comorbidity, and cancer stage. Findings suggest that TACE with or without sorafenib is currently the most cost-effective active non-curative palliative treatment approach to HCC. Further research into new combination treatment strategies that afford the best tumor response is needed.

## Introduction

Liver cancer is the sixth most common cancer and the second leading cause of cancer-related death worldwide [1]. In Canada, hepatocellular carcinoma (HCC) incidence continues to rise and five-year relative survival rates remain poor (~20%) [2]. HCC accounts for the majority (~72%) of primary liver cancers in Canada [2]. Risk factors for HCC include cirrhosis, chronic hepatitis B or C infection, HIV co-infection, alcoholic- and non-alcoholic fatty liver disease, diabetes, obesity, and smoking [3,4].

Transarterial chemoembolization (TACE) is the standard of care for patients with intermediate-stage disease [5,6], and survival times and time to progression appear longer in patients with the combination of sorafenib and TACE [7–9]. Sorafenib, a multikinase inhibitor, as an oral form of systemic therapy for patients with advanced HCC has shown improved survival and time to progression in patients with advanced HCC; however, its use remains substantial financial burden [10–12]. There is mixed evidence regarding the cost-effectiveness of sorafenib using decision analytic Markov models. Studies using data from the SHARP trial [10] determined that sorafenib is cost-effective compared to best supportive care (BSC) with an incremental cost-effectiveness ratio (ICER) within the established willingness-to-pay threshold between $50,000 and $100,000/life year (LY) gained [13,14]. The Italian SOFIA study concluded that dose-adjusted sorafenib is cost-effective compared to BSC in intermediate and advanced HCC with an ICER of less than a threshold of €38,000/quality-adjusted life year (QALY) gained (~$50,000/QALY) [15]. Though, another study found that sorafenib is not a cost-effective treatment option for Chinese patients with advanced HCC [16].

Historically, HCC was diagnosed after developing symptoms which correlates with advanced-stage disease, limited therapeutic options, and poor prognosis [3,7]. Patients with a poor prognosis often undergo non-curative palliative treatments, including TACE, sorafenib, chemotherapy, or purely supportive care. The decision to pursue any of the aforementioned therapies is multifactorial and a systematic comparison of the financial impact of these interventions has not been done.

In this study, we aim to evaluate the cost-effectiveness of alternative non-curative palliative treatment interventions of TACE alone or TACE+sorafenib, sorafenib alone, and non-sorafenib chemotherapy alone compared with no treatment or BSC (i.e. symptom management to improve quality of life but no specific antineoplastic therapy) using person-level data from the Canadian health care perspective. Our cost-effectiveness analysis uses a net benefit regression approach and accounts for the fact that patients do not randomly receive non-curative palliative treatments in the real-world. Results from this study will examine the utility of TACE alone or TACE and sorafenib combination therapy as an anticancer agent and help inform health policy regarding the treatment of intermediate or advanced HCC.

## Materials and methods

### Study design and population

We identified all eligible HCC cases aged 18 years and older in Ontario diagnosed between January 1, 2007 and December 31, 2010. These HCC cases were used to estimate the cost, effectiveness, and cost-effectiveness of alternative non-curative palliative treatment strategies compared with no treatment or BSC to provide an estimate of the trade-off between extra cost and extra benefit as well as utilizing net benefit regression framework to estimate the incremental net benefit (INB). HCC cases were identified through the Ontario Cancer Registry (OCR). The International Statistical Classification of Disease and Related Health Problems, 9th Revision (ICD-9) site code 155.0, in combination with histology codes 8170–8175 of the International Classification of Diseases for Oncology, Third Edition (ICD-O-3) were used to identify cases of primary liver cancer. Cases of primary liver cancer were identified only using ICD-9 coding due to the lack of ICD-10 C22 code in the dataset. Patients who had death dates before or on the HCC diagnosis date during the study period were excluded. Furthermore, potential curative treatments such as radiofrequency ablation, surgical resection, liver transplantation, and percutaneous ethanol injection for small HCC or unresectable liver cancer treatment were also excluded.

### Data sources and study variables

The OCR is a provincial population-based cancer registry that contains information on all new cases of cancer (except for non-melanoma skin cancers) in Ontario since 1964 [17]. The OCR includes data for the date and stage of HCC diagnosis, age, sex, birth location, urban or rural residence, cause of death, and date of death. As in previous studies [18–21], we linked the OCR cohort to the Ontario Health Insurance Plan (OHIP) database, the Canadian Institute for Health Information Discharge Abstract Database, the Ontario Drug Benefit (ODB) program database, and the Canadian census data to provide person-level information on sociodemographic, screening, staging, treatment, and clinical factors. The OHIP physician billing claims contain service and diagnosis information for outpatient visits. The Discharge Abstract Database contains information relating to in-hospital procedures and diagnoses. The ODB dataset contains information regarding prescription medications (including sorafenib) dispensed to all adults aged 65 years and older and those receiving social assistance. Although there are some variances in different health care services, the system provides free access to hospital and emergency department visits, physician services, homecare, co-payments for long-term care placements, and prescription medications for those aged 65 years and older.

Area-level socio-economic status was quantified using median neighbourhood household income. Median neighbourhood household income was determined through linking of postal codes to Canadian census data; income was categorized into quintiles corresponding to income status of neighbourhoods. The income quintile 1 represents the lowest 20% of neighborhoods and income quintile 5 represents the most well-off 20% of neighbourhoods [18].

Where possible, hospitalization records from the date of diagnosis were used to assign each patient and control subject a baseline Charlson–Deyo comorbidity index. If patients did not have a hospitalization record at their diagnosis date, baseline comorbidity was determined by looking back 2 years into the hospitalization data to find the most recent hospitalization record; the comorbidity score from that hospitalization was then applied [18–21]. The Charlson–Deyo comorbidity index at baseline was marked as “missing” if the individual had no hospitalization records at diagnosis or during the 2 years before diagnosis. Comorbidity was adjusted for each hospitalization after baseline. The Charlson–Deyo comorbidity index was calculated using methods previously described [22,23]; an ICD-10 coding algorithm was applied to the diagnostic field codes from the hospitalization data (excluding diagnoses for liver disease, metastatic cancer, diabetes, and HIV). Conditions were weighted and then summed up to provide an overall comorbidity index value for a given episode, which was then categorized into one of five groups (0, 1, 2, ≥ 3, or no hospitalization record) representing different degrees of comorbidity.

Patients diagnosed with diabetes, HIV, and covariates that denote liver disease stage measured before HCC diagnosis were identified from the Discharge Abstract Database and OHIP using ICD-9 and ICD-10 codes. The study also included viral hepatitis cases identified through OHIP data; defined as subjects having at least two viral hepatitis visits (OHIP diagnostic code ‘070’) within the 4-year interval before the HCC diagnosis date ─ to cover as much available OHIP data as possible. Indicators of liver disease stage were categorized exclusively as: 1) viral hepatitis; 2) no cirrhosis; 3) cirrhosis; 4) alcoholic liver disease (ALD)+cirrhosis; 5) viral hepatitis+cirrhosis; 6) decompensated cirrhosis (i.e. cirrhosis and any recorded ascites, esophageal varices, or hepatic encephalopathy); 7) ALD+decompensated cirrhosis; 8) viral hepatitis+decompensated cirrhosis; and 9) ALD+viral hepatitis+decompensated cirrhosis.

To identify patients who received screening ultrasonography, we identified all abdominal ultrasonography performed on patients before HCC diagnosis utilizing OHIP fee codes [20]. We obtained exclusive data regarding receipt of abdominal ultrasound screening (at least 4.5 months apart from previous ultrasound), which was defined as receiving one or more ultrasound screening annually for 2 years before HCC diagnosis (i.e. routine screening), at least one screen either within 12 months or between 12–24 months before HCC diagnosis (i.e. inconsistent screening), and no screening before HCC diagnosis.

Classification of malignant tumors based on TNM staging [extent of the tumor (T), extent of spread to the lymph nodes (N), and presence of metastasis (M)] [24] was used in the OCR from 2004 onwards. TACE, non-sorafenib chemotherapy, and BSC were identified from the OHIP database and sorafenib was identified from the ODB database.

### HCC treatment strategies

Mutually exclusive non-curative palliative treatment for HCC considered over the study period include: i) TACE alone or TACE+sorafenib; ii) sorafenib alone; iii) non-sorafenib chemotherapy alone; and iv) no treatment or BSC. The initial date of the first non-curative palliative treatment was considered the index date of treatment for HCC patients. Procedure codes used to identify diabetes, HIV, indicators of liver disease stage, HCC screening, and treatments can be found elsewhere [20,21].

### Measuring effectiveness

Life expectancy for each age is the average period that a person may expect to live, according to the age-specific mortality rates for all causes [25]. LYs, QALYs, potential years of life lost (PYLL, a measure of premature mortality) and quality-adjusted life years lost (QALYL) were used to measure effectiveness. This study followed patients according to their death status until the end of year 2011. For those who died in or before 2011, age at death was calculated by adding years between diagnosis and death to the age at diagnosis. Age at diagnosis was recorded in the OCR data as a categorical variable: below 60, 60–69, 70–79, or 80 years and above, which was assumed to be 55, 65, 75, or 85 years, respectively in our analysis. To estimate age at death for patients who were still alive by the end of 2011, we first calculated the expected year of death using the year of diagnosis and the expected length of survival (i.e. period from diagnosis to death) according to stage at diagnosis from the published literature (e.g. early-stage I: 5 years; intermediate-stage II: 4 years; and advanced-stage III or IV: 3 years survival) [26,27]. If the expected year of death was 2011 or earlier, given the patient was still alive by the end of 2011, we assumed 2012 to be the most likely year of death. Accordingly, age at death could be estimated based on age at diagnosis and years between death and diagnosis. Subsequently, PYLL for each patient was determined using Ontario life tables which provided the standard life expectancy based on sex and age at death of an individual person [28].

QALYL consisted of two parts: 1) PYLL was weighted by the average health state utility should the person be still alive and without disease; and 2) number of years between diagnosis and death weighted by the quality of life according to stage of cancer (from normal utility to utility of HCC stage I, II, III, IV). Although we developed the year-specific model and considered treating stage as time-dependent, only stage at diagnosis was available in the database; we could not obtain whether patients progressed beyond their disease stage at diagnosis. Mean health state utilities of HCC by stage were derived from published literature and assumption for base case analysis and the lower and upper bounds for sensitivity analyses are shown in the S1 Table.

### Measuring costs

Full details of data sources and estimation of direct health care costs associated with HCC have been previously published [19]. The total costs of health care services included outpatient visits, emergency department visits, acute inpatient hospitalizations, same-day surgery, prescription medications, homecare visits, continuing care, and long-term care. Costs associated with outpatient physician visits and laboratory tests in Ontario were estimated from the Physicians Claims History Database of the OHIP. Costs for emergency department visits and same-day surgery were estimated using the National Ambulatory Care Reporting System database [29]. Costs of hospitalization, emergency department visits, and same-day surgery for a particular year were estimated using the Resource Intensity Weight methodology developed by the Canadian Institute for Health Information [29–31]. Prescription medication costs were obtained from the ODB Program [29]. Costs associated with home care, continuing care, and long-term care were estimated from the Ontario Home Care database, Continuing Care Reporting System, and ODB Program. Costs were adjusted for inflation to 2013 Canadian dollars using the Statistics Canada Consumer Price Index for health care and personal items for Ontario [32]. Purchasing Power Parity for Gross Domestic Product was used to convert 2013 Canadian dollars to 2013 U.S. dollars [33]. Effects and costs were discounted at 3% annually as a base case to capture time preference given variation in follow-up time [34].

### Statistical analysis

The net benefit regression framework [35] was used to evaluate the real-world cost-effectiveness of alternative non-curative palliative treatment strategies compared with no treatment or BSC among patients diagnosed with HCC from 2007 to 2010. In the first step, the net benefit value for each person (*NB*_*i*_) was calculated using the formula: willingness-to-pay threshold (*λ*)*E_*i*_ ‒ *C*_*i*_, where *E*_*i*_ is the observed incremental effect (i.e. LY or QALY gained) and *C*_*i*_ is the incremental cost, for the *i*th person. Various values of *λ* for an additional effect were explored ranging from $0 to $500,000. *NB*_*i*_ differs by various levels of *λ*; therefore, the person-level net benefit is denoted as *NB(λ)*_*i*_.

The net benefit regression (i.e. multiple linear regression) involved fitting a linear regression model while adjusting for the relevant covariates (dummy variables), including sociodemographic characteristics: age (< 60, 60–69, 70–79, ≥ 80 years); sex (male, female); income quintile (Q1-lowest to Q5-highest); residence (urban, rural); birth country (Canada, outside of Canada); clinical characteristics: Charlson-Deyo comorbidity index (0, 1, 2, ≥ 3); diabetes; HIV; liver disease stage (i.e. viral hepatitis; no cirrhosis; cirrhosis; ALD+cirrhosis; viral hepatitis+cirrhosis; decompensated cirrhosis; ALD+decompensated cirrhosis; viral hepatitis+decompensated cirrhosis; and ALD+viral hepatitis+decompensated cirrhosis); receipt of ultrasound screening 2 years before HCC diagnosis (routine screening, inconsistent screening, no screening); stage at diagnosis (early-stage I, intermediate-stage II, advanced-stage III-IV); and index year of HCC diagnosis (2007, 2008, 2009, 2010). Additionally, we adjusted for propensity score to minimize bias related to the non-random allocation of palliative treatment [36,37]. The propensity score for an individual is the conditional probability of assignment to having a palliative treatment of HCC given the observed individual covariates. Here, it was derived by fitting a logistic regression model with HCC non-curative palliative treatment as the dependent variable and the aforementioned covariates as independent variables. This approach allows the adjustment of how covariates may affect the estimate of the intervention’s INB (i.e. the marginal impact on ICER) [35]. To examine this, we employed an empirical model that interacts three treatment dummy variables with the covariates as follows:

$$NB({\lambda )}_{i}=\alpha +\sum_{j=1}^{n}\beta_{j}x_{ij}+\delta_{1}T_{1i\ldots \ldots .}+\delta_{3}T_{3i}+\sum_{j=1}^{n}\gamma_{j}{T_{i}x}_{ij}+\varepsilon_{i}$$

where: NB(λ)_i_ is the person-level NB; α is an intercept term; T_i_ is a treatment dummy, indicating whether person i received treatment (i.e. T_i_ = 1) or no treatment (i.e. T_i_ = 0); β is the coefficient estimate for the aforementioned covariates of interest x; γ is an interaction term between subject characteristic and the treatment indicator; and ε is a stochastic error term assumed to be normally distributed. The regression coefficient δ on the treatment dummy provides the estimate of the INB of treatment versus no treatment corresponding to a certain level of *λ* adjusted for the covariates. Treatment is defined to be cost-effective, at a certain level of *λ*, if the corresponding INB is positive (i.e. INB > 0). The INB was displayed visually by plotting the INB and its 95% confidence intervals (CIs) over the range of willingness-to-pay values.

Threshold values of the variance inflation factors were evaluated in the context of several other factors that influence the variance of regression coefficients [38]. We eliminated interaction terms if there was no statistical significance or if the variance inflation factor values exceeded 10 (i.e. indicating severe multicollinearity), which can reduce the variance of the regression coefficients. All covariates were included in the model because they were considered to be significant correlates of the outcome (theoretical justification) or were significantly different between the treatments (statistical validation). The final net benefit model comprised of:

$$\begin{array}{l} NB_{i}=\alpha +\delta_{1}{\left(T_{1}\right)}_{i}\ldots \ldots +\delta_{3}{\left(T_{3}\right)}_{i}+\beta_{1}{\left(age\right)}_{i}+\beta_{2}{\left(sex\right)}_{i}+\beta_{3}{\left(income\;quintile\right)}_{i}+\beta_{4}(urban\;or\;rural\\ residence{)}_{i}+\beta_{5}{\left(birth\;country\right)}_{i}+\beta_{6}{\left(Charlson\text{-}Deyo\;comorbidity\;index\right)}_{i}+\beta_{7}{\left(diabetes\right)}_{i}+\beta_{8}{\left(HIV\right)}_{i}+\\ \beta_{9}{\left(indicators\;of\;liver\;disease\;stage\right)}_{i}+\beta_{10}{\left(ultrasound\;screening\right)}_{i}+\beta_{11}{\left(stage\;at\;HCC\;diagnosis\right)}_{i}+\\ \beta_{12}{\left(index\;year\right)}_{i}+\beta_{13}{\left(propensity\;score\right)}_{i}+\varepsilon_{i} \end{array}$$

The final step was assessing uncertainties and constructing cost-effectiveness acceptability curves (CEACs) using the coefficient estimates of the treatment (*T*) variable and *p*-values obtained from the net benefit regression model [39–42]. A CEAC shows the probability that an intervention is cost-effective compared with the alternative, over a range of threshold values that decision makers may be willing-to-pay for an additional unit of LY or QALY [35].

### Sensitivity analysis

Lower and upper bounds health state utilities of HCC by stage (±25%) were used for cost-effectiveness sensitivity analyses (S1 Table). In addition, pooled mean health state utilities by liver disease stage (S2–S5 Tables) [43–51], health state utilities for incurable HCC (mean 0.40) [52] or after disease progression (0.68) [53] from published literature were used for cost-effectiveness sensitivity analyses. In addition, multiple imputation was used to impute values for variables with a significant portion of missing data. Variables which were imputed were income quintile (n = 6, 0.5%), birth country (n = 98, 8.4%), Charlson-Deyo comorbidity index (n = 261, 22.3%), and cancer stage at HCC diagnosis (n = 510, 43.5%). The observed important covariates considered were age, sex, index year of HCC diagnosis, and ultrasound screening. Five independent draws from an imputation model were used to create five completed data sets and results were combined to obtain one imputation inference. Multiple Imputation procedure by logistic regression was used in a sequential process to generate monotone patterns (PROC MI with LOGISTIC in the MONOTONE statement) [54].

Analyses were performed using the SAS (version 9.4: SAS Institute, Cary, NC, USA) and the STATA (version 12.0: Stata Corporation, College Station, TX) statistical software applications.

### Ethics approval

Ethics approval for the study was granted by the University of Toronto Health Sciences Research Ethics Board. Informed consent was not obtained because this secondary analysis accessed existing de-identified data; consent was therefore deemed to be neither feasible nor necessary by the Research Ethics Board.

## Results

### Description of cohort

Overall, 2,012 patients were identified as having a primary diagnosis of HCC from the OCR between 2007 and 2010. A representative flow chart of the study population can be found in S1 Fig. The final study cohort comprised 1,172 patients diagnosed with HCC after excluding patients who had curative treatments (radiofrequency ablation, surgical resection, liver transplantation, and percutaneous ethanol injection; n = 784) and relatively small number of non-curative palliative treatments (i.e. sorafenib+chemotherapy, TACE+chemotherapy, and TACE+sorafenib+chemotherapy; n = 56). The median and mean follow-up time of patients diagnosed with HCC were 152.5 days and 304 (standard deviation 377) days, respectively. Overall baseline characteristics for this cohort are summarized in S6 Table and those stratified by treatment are summarized in Table 1. Fifty three (4.5%) patients diagnosed with HCC received TACE alone (n = 38) or TACE+sorafenib (n = 15), 93 (7.9%) patients received sorafenib alone, and 66 (5.6%) patients received non-sorafenib chemotherapy alone during the study period; however, 960 (81.9%) patients received BSC or did not receive any treatment. With regard to TACE+sorafenib dual treatments, TACE was the first-line treatment (100%) anywhere from 100 to 1605 days before receiving sorafenib. Of 1,172 patients, 5.7% were stage I, 9.0% stage II, 28.5% stage III, 13.2% stage IV, and 43.5% unknown stage at diagnosis (S6 Table). No cirrhosis or cirrhosis and cancer stage were associated with receipt of all types of treatments (*P* < 0.05); Urban/rural residence and comorbidity were associated with TACE alone or TACE+sorafenib treatment and chemotherapy alone (*P* < 0.05). Birth country and ultrasound screening were associated with TACE alone or TACE+sorafenib treatment (*P* < 0.001). Additionally, age group and year of HCC diagnosis were associated with sorafenib treatment (*P* < 0.01). Patients with unknown stage were less likely to have received non-curative palliative treatments.

**Table 1:** Baseline characteristics of patients with hepatocellular carcinoma by type of treatment, 2007–2010.

	No treatment or BSC	TACE alone or TACE + Sorafenib	Sorafenib alone	Non-sorafenib chemotherapy alone
	n (%)	n (%)	n (%)	n (%)
Overall	960 (81.9)	53 (4.5)	93 (7.9)	66 (5.6)
Age group (years)				
<60	269 (28.0)	13 (24.5)	16 (17.2)	25 (37.9)
60–69	235 (24.5)	14 (26.4)	24 (25.8)	18 (27.3)
70–79	271 (28.2)	19 (35.9)	41 (44.1)	17 (25.8)
80+	185 (19.3)	7 (13.2)	12 (12.9)	6 (9.1)
Sex				
Female	207 (21.6)	13 (24.5)	15 (16.1)	13 (19.7)
Male	753 (78.4)	40 (75.5)	78 (83.9)	53 (80.3)
Income quintile				
Q1 (lowest)	273 (28.4)	8 (15.1)	22 (23.7)	15 (22.7)
Q2	224 (23.3)	10 (18.9)	22 (23.7)	18 (27.3)
Q3	145 (15.1)	15 (28.3)	11 (11.8)	12 (18.2)
Q4	150 (15.6)	9 (17.0)	18 (19.4)	11 (16.7)
Q5 (highest)	163 (17.0)	11 (20.8)	20 (21.5)	9 (13.6)
Missing	-	0	0	-
Residence				
Urban	861 (89.7)	50 (94.3)	84 (90.3)	52 (78.8)
Rural	98 (10.2)	3 (5.7)	9 (9.7)	14 (21.2)
Missing	-	0	0	0
Birth country				
Canada	476 (49.6)	14 (26.4)	34 (36.6)	40 (60.6)
Other	413 (43.0)	28 (52.8)	48 (51.6)	21 (31.8)
Unknown/Missing	71 (7.4)	11 (20.8)	11 (11.8)	-
Charlson-Deyo comorbidity index				
0	315 (32.8)	22 (41.5)	37 (39.8)	27 (40.9)
1	239 (24.9)	21 (39.6)	29 (31.2)	15 (22.7)
2	102 (10.6)	7 (13.2)	7 (7.5)	14 (21.2)
3+	62 (6.5)	-	6 (6.5)	-
No hospitalization record	242 (25.2)	0	14 (15.1)	-
Diabetes diagnosis	475 (49.5)	21 (39.6)	50 (53.8)	27 (40.9)
HIV	15 (1.6)	-	-	-
Indicators of liver disease stage				
Viral hepatitis	29 (3.0)	-	-	-
No cirrhosis	245 (25.5)	-	37 (39.8)	27 (40.9)
Cirrhosis	159 (16.6)	17 (32.1)	14 (15.1)	-
ALD + Cirrhosis	29 (3.0)	-	-	-
Viral hepatitis + Cirrhosis	29 (3.0)	-	-	-
Decompensated cirrhosis	245 (25.5)	17 (32.1)	22 (23.7)	14 (21.2)
ALD + Decompensated cirrhosis	137 (14.3)	-	-	7 (10.6)
Viral hepatitis + Decompensated cirrhosis	28 (2.9)	-	-	-
ALD + Viral Hepatitis + Decompensated cirrhosis	28 (2.9)	-	0	0
Ultrasound screening 2 years before HCC diagnosis				
≥1 screens annually	57 (5.9)	11 (20.8)	-	7 (10.6)
Inconsistent screening	351 (36.6)	21 (39.6)	30 (32.3)	23 (34.9)
No screening	552 (57.5)	21 (39.6)	58 (62.4)	36 (54.6)
Stage at HCC diagnosis				
Early (stage I)	51 (5.3)	12 (22.6)	-	-
Intermediate (stage II)	69 (7.2)	14 (26.4)	10 (10.8)	13 (19.7)
Advanced (stage III-IV)	372 (38.8)	20 (37.7)	64 (68.8)	33 (50.0)
Unknown	468 (48.8)	7 (13.2)	17 (18.3)	18 (27.3)
Year of HCC diagnosis				
2007	248 (25.8)	13 (24.5)	8 (8.6)	14 (21.2)
2008	210 (21.9)	14 (26.4)	17 (18.3)	16 (24.2)
2009	233 (24.3)	13 (24.5)	35 (37.6)	23 (34.9)
2010	269 (28.0)	13 (24.5)	33 (35.5)	13 (19.7)

n = 1,172.

‘‘-“, counts less than six have been suppressed.

TACE, transarterial chemoembolization; BSC, best supportive care (formal palliative care); HCC, hepatocellular carcinoma.

### Health care effects and costs

Effects and costs after diagnosis of HCC stratified by treatment strategies are summarized in Table 2. The lowest QALYL was among those who received sorafenib alone (9.77, 95% CI: 9.01–10.53) and the highest QALYL was among those who received non-sorafenib chemotherapy alone (11.57, 95% CI: 10.58–12.57). The lowest costs were among those who did not receive treatment or BSC ($36,415, 95% CI: $33,782-$39,048), followed by those who received TACE alone or TACE+sorafenib ($45,638, 95% CI: $39,180-$52,096); and the highest cost was among those who received sorafenib alone ($53,198, 95% CI: $44,941-$61,456), followed by those who received chemotherapy alone ($51,657, 95% CI: $38,913-$64,402).

**Table 2:** Health care effects and costs after diagnosis of hepatocellular carcinoma by treatment strategies, 2007–2010.

Treatment strategies	Effects (mean, 95% CI)		Costs* (mean, 95% CI)
	PYLL	QALYL	
No Treatment or BSC (n = 960)	11.5710 (11.2764–11.8655)	10.6226 (10.3531–10.8921)	$36,415 ($33,782-$39,048)
TACE alone or TACE + Sorafenib (n = 53)	10.7860 (9.5982–11.9739)	10.0879 (9.0078–11.1680)	$45,638 ($39,180-$52,096)
Non-sorafenib chemotherapy alone (n = 66)	12.4255 (11.3347–13.5163)	11.5722 (10.5770–12.5675)	$51,657 ($38,913-$64,402)
Sorafenib alone (n = 93)	10.4988 (9.6655–11.3320)	9.7664 (9.0062–10.5266)	$53,198 ($44,941-$61,456)

*All costs reflect 2013 US$ per person.

BSC, best supportive care; TACE, transarterial chemoembolization; CI, confidence intervals; PYLL, potential years of life lost (a measure of premature mortality); QALYL, quality-adjusted life years lost.

### Net benefit regression

**Fig 1:**
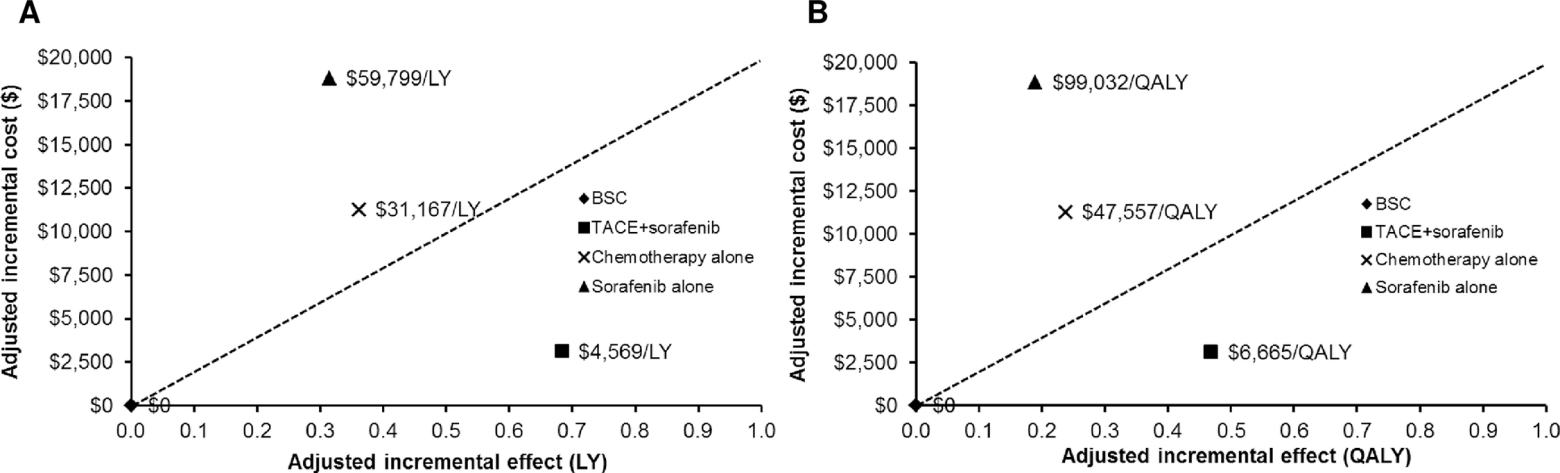
**A and B**. Efficiency frontier: plot of incremental (A) life years (LYs) and (B) quality-adjusted life years (QALYs) and costs of non-curative palliative treatments: i) transarterial chemoembolization (TACE) alone or TACE+sorafenib; ii) non-sorafenib chemotherapy alone; and iii) sorafenib alone relative to lowest cost scenario (no treatment or best supportive care [BSC]). The dotted diagonal line represents the ceiling ratio. If an intervention lies above the line, it will not be acceptable on cost-effectiveness grounds.

Compared with no treatment or BSC (adjusted for important covariates), TACE alone or TACE+sorafenib was estimated to yield the highest incremental QALYs (incremental QALYs = 0.47), followed by chemotherapy alone (0.24) and sorafenib alone (0.19). Among treatments, TACE alone or TACE+sorafenib was more effective and slightly more costly than no treatment or BSC (Table 3). Fig 1A and 1B demonstrate plots of incremental effects (LYs and QALYs) and incremental costs of treatments relative to the lowest cost scenario (no treatment or BSC), TACE alone or TACE+sorafenib treatments below the line (dotted diagonal line representing the ceiling ratio) appeared to be acceptable.

**Table 3:** Adjusted incremental effects, incremental costs, and incremental cost-effectiveness ratios of non-curative palliative treatment strategies for hepatocellular carcinoma compared with no treatment or best supportive care, 2007–2010: net benefit regression.

Treatment Strategies	Mean LYs	Mean QALYs	Mean Total Effect (PYLL)	Mean Total Effect (QALYL)	MeanTotal Cost ($)	Adjusted Incremental Effect* (LYs)	Adjusted Incremental Effect* (QALYs)	Adjusted Incremental Cost ($)^†^	Adjusted ICER ($/LY gained)	Adjusted ICER ($/QALY gained)
No treatment or BSC	0.7034	0.5422	11.5710	10.6226	$36,415					
TACE alone or TACE + Sorafenib	1.6715	1.2828	10.7860	10.0879	$45,638	0.68283	0.46815	$3,120	$4,569	$6,665
Non-sorafenib chemotherapy alone	1.3314	0.9628	12.4255	11.5722	$51,657	0.36137	0.23683	$11,263	$31,167	$47,557
Sorafenib alone	1.3370	0.9474	10.4988	9.7664	$53,198	0.31474	0.19005	$18,821	$59,799	$99,032

*Incremental effect is calculated as treatment effect minus no treatment or BSC effect, adjusted for relevant covariates (dummy variables), including age, sex, income quintile, urban/rural residence, birth country, Charlson-Deyo comorbidity index, diabetes, HIV, indicators of liver disease stage, ultrasound screening, stage at HCC diagnosis, and year of HCC diagnosis. Positive value indicates increase in the effect relative to “no treatment or BSC”.

^†^Incremental cost is calculated as treatment cost minus no treatment or BSC cost, adjusted for aforementioned covariates. Positive value indicates increase in cost relative to “no treatment or BSC”. Values are expressed as the mean. All costs reflect 2013 US$ per person.

BSC, best supportive care (formal palliative care); TACE, transarterial chemoembolization; PYLL, potential years of life lost; QALYL, quality-adjusted life years lost; LY, life year; QALY, quality-adjusted life years.

**Fig 2:**
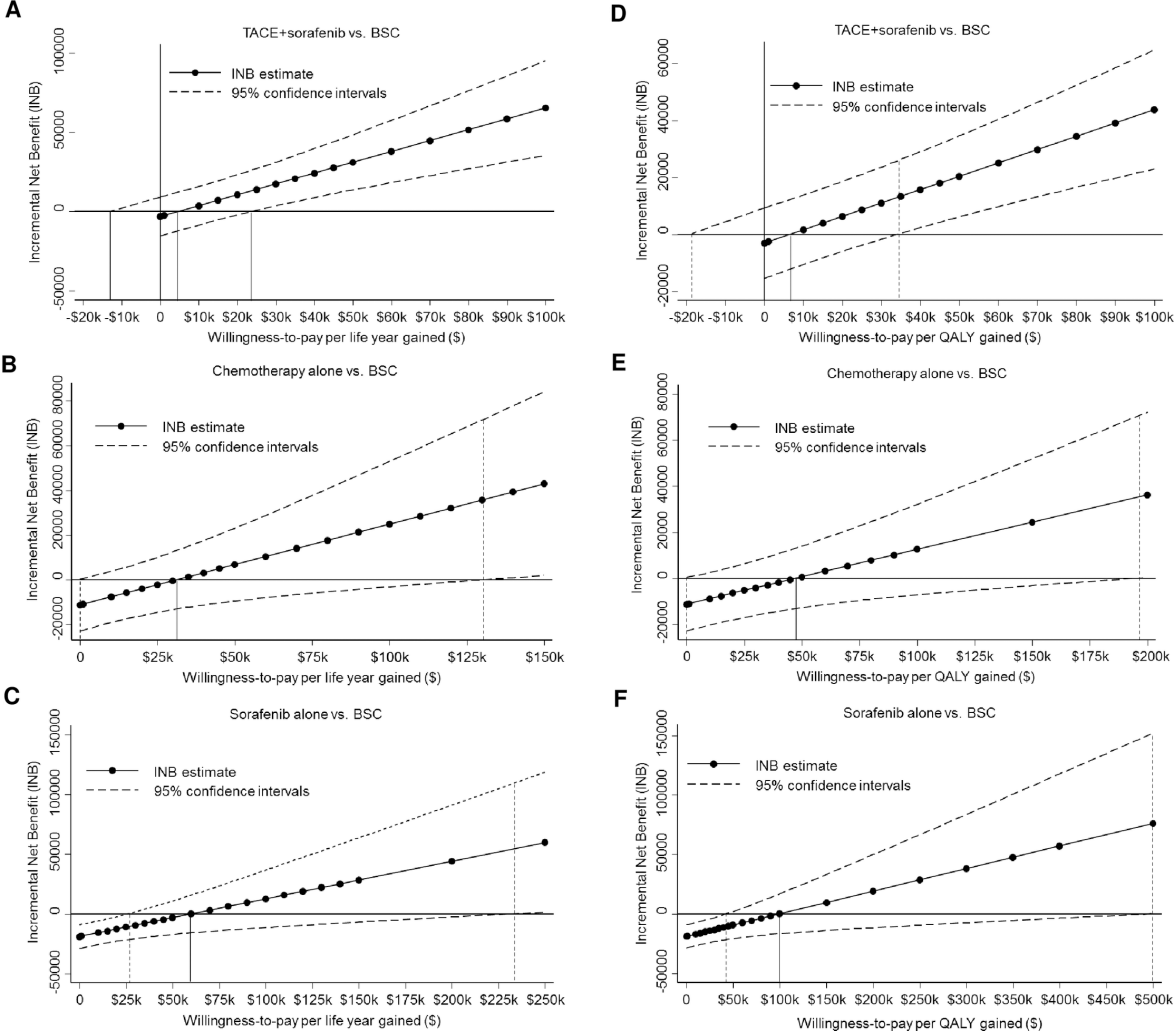
**A-F**. Estimates of incremental net benefit (i.e. incremental cost-effectiveness ratio, ICER) and its 95% confidence intervals as a function of willingness-to-pay threshold for an additional life year: (A) transarterial chemoembolization (TACE) alone or TACE+sorafenib vs. no treatment or best supportive care (BSC); (B) non-sorafenib chemotherapy alone vs. BSC; and (C) sorafenib alone vs. BSC; and for an additional quality-adjusted life year (QALY): (D) TACE alone or TACE+sorafenib vs. BSC; (E) non-sorafenib chemotherapy alone vs. BSC; and (F) sorafenib alone vs. BSC.

Fig 2A–2C (LYs) and Fig 2D–2F (QALYs) show estimates of INB (i.e. ICER estimate) and its 95% CIs as a function of willingness-to-pay threshold. The lowest ICER estimates for TACE alone or TACE+sorafenib were $6,665/QALY gained (95% CI: -$18,800-$34,500/QALY). Alternative ICER estimates in order were: for chemotherapy, $47,557/QALY (95% CI: $0-$196,500/QALY); and sorafenib alone, $99,032/QALY (95% CI: $42,500-$500,000/QALY).

**Fig 3:**
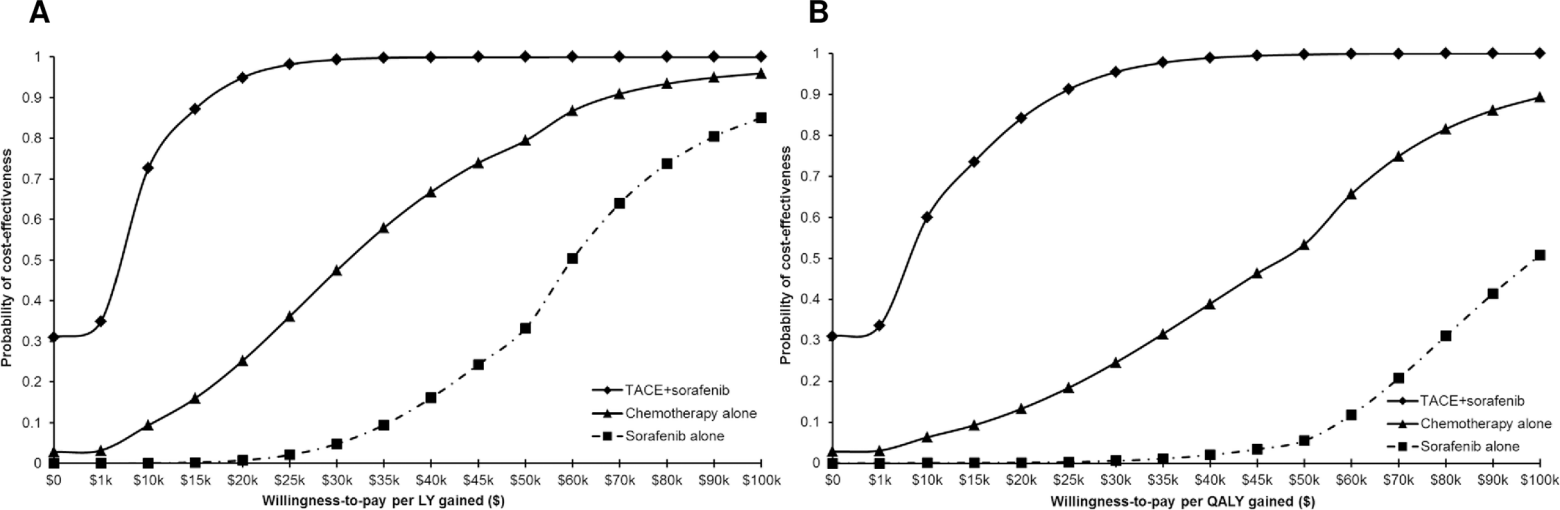
**A and B**. Cost-effectiveness acceptability curves showing the probability that each non-curative palliative treatment strategy: i) TACE alone or TACE+sorafenib; ii) non-sorafenib chemotherapy alone; or iii) sorafenib alone is cost-effective compared with no treatment or BSC for a given willingness-to-pay threshold for an additional (A) life year (LY); and (B) quality adjusted life year (QALY).

Fig 3A and 3B show CEACs which plot the probability that each treatment strategy is cost-effective compared with no treatment or BSC as a function of willingness-to-pay threshold for an additional LY and QALY, respectively (S7 Table and Table 4). The results showed that if with a threshold of $50,000/QALY gained, TACE alone or TACE+sorafenib treatments would have a cost-effectiveness probability of 99.7%; chemotherapy and sorafenib treatments would have a cost-effectiveness probability of 53.4% and 5.5%, respectively (Fig 3B and Table 4). If a threshold of $100,000/QALY gained was to be chosen, TACE alone or TACE+sorafenib treatments would have a cost-effectiveness probability of 100%, and chemotherapy and sorafenib alone would have a cost-effectiveness probability of 89.3% and 50.9%, respectively (Fig 3B and Table 4).

**Table 4:** Estimates of incremental net benefit and probability of cost-effectiveness of non-curative palliative treatment strategies for hepatocellular carcinoma compared with no treatment or best supportive care as a function of willingness-to-pay threshold per additional QALY over the study period 2007–2010.

λ thresholds	TACE alone or TACE + Sorafenib			Sorafenib alone			Non-sorafenib chemotherapy alone		
	INB estimate (SE)	*P*-value*	Probability of cost-effectiveness	INB estimate (SE)	*P*-value*	Probability of cost-effectiveness	INB estimate (SE)	*P*-value*	Probability of cost-effectiveness
$0	-3120 (6284)	0.310	31.0%	-18821 (5049)	<0.001	0%	-11263 (5898)	0.028	2.8%
$1,000	-2652 (6267)	0.336	33.6%	-18631 (5035)	<0.001	0%	-11026 (5883)	0.031	3.1%
$10,000	1561 (6183)	0.400	60.0%	-16920 (4968)	<0.001	0%	-8895 (5804)	0.063	6.3%
$20,000	6243 (6236)	0.159	84.1%	-15020 (5010)	0.001	0.2%	-6526 (5853)	0.133	13.3%
$30,000	10924 (6438)	0.045	95.5%	-13119 (5172)	0.006	0.6%	-4158 (6043)	0.246	24.6%
$40,000	15606 (6776)	0.011	98.9%	-11219 (5444)	0.020	2.0%	-1790 (6361)	0.389	38.9%
$50,000	20288 (7232)	0.003	99.7%	-9318 (5810)	0.055	5.5%	579 (6788)	0.466	53.4%
$60,000	24969 (7784)	0.001	99.9%	-7418 (6254)	0.118	11.8%	2947 (7306)	0.343	65.7%
$70,000	29651 (8413)	<0.001	100%	-5517 (6760)	0.207	20.7%	5315 (7897)	0.251	74.9%
$80,000	34332 (9104)	<0.001	100%	-3617 (7315)	0.311	31.1%	7683 (8546)	0.185	81.6%
$90,000	39014 (9844)	<0.001	100%	-1716 (7909)	0.414	41.4%	10052 (9241)	0.139	86.1%
$100,000	43695 (10622)	<0.001	100%	184 (8534)	0.491	50.9%	12420 (9971)	0.107	89.3%

*one-sided *P*-value.

TACE, transarterial chemoembolization; BSC, best supportive care (formal palliative care); QALY, quality-adjusted life year; λ, willingness-to-pay; INB, incremental net benefit; SE, standard error.

### Sensitivity analysis

Plots of incremental effects (QALYs) and incremental costs of treatments relative to the lowest cost scenario (no treatment or BSC) according to the lower (-25%) and upper bound (+25%) health state utilities of cancer stage showed that TACE alone or TACE+sorafenib treatments appeared to be acceptable, similar to base case (S2 Fig). Similarly, when using pooled mean health state utilities by liver disease stage, health state utilities for incurable HCC or after disease progression, TACE alone or TACE+sorafenib was found to be the lowest ICER estimates $4,152/QALY gained (95% CI: -$11,050-$20,900/QALY), followed by chemotherapy alone, $28,291/QALY (95% CI: $0-$70,000/QALY) and sorafenib alone, $50,569/QALY (95% CI: $23,800-$96,800/QALY) (S3–S5 Figs).After multiple imputation for variables of missing data such as income quintile, birth country, Charlson-Deyo comorbidity index, and cancer stage at EAC diagnosis and adjusting for confounding covariates, plots of incremental effects and costs of treatments relative to no treatment or BSC, TACE alone or TACE+sorafenib treatments appeared to be acceptable (S6 Fig). Additionally, ICER estimates in order were for: TACE alone or TACE+sorafenib, $16,206/QALY gained (95% CI: -$5,800-$45,000/QALY); non-sorafenib chemotherapy alone, $47,881/QALY (95% CI: $12,500-$153,000/QALY); and sorafenib alone, $75,128/QALY (95% CI: $37,500-$205,500/QALY) (S7 Fig). The CEACs showed that if a threshold of $50,000/QALY gained was to be chosen, TACE alone or TACE+sorafenib treatments would have a cost-effectiveness probability of 98.7%; non-sorafenib chemotherapy and sorafenib alone would have a cost-effectiveness probability of 53.8% and 12.3%, respectively. If a threshold of $100,000/QALY gained was to be chosen, TACE alone or TACE+sorafenib treatments would have a cost-effectiveness probability of 100%, and chemotherapy and sorafenib alone would have a cost-effectiveness probability of 92.3% and 76.5%, respectively (S8 Fig).

After multiple imputation, when TACE alone was used as a separate treatment, TACE+sorafenib was estimated to yield the highest adjusted incremental QALYs (0.80), followed by TACE alone (0.37), non-sorafenib chemotherapy (0.29), and sorafenib alone (0.26) compared with no treatment or BSC. In contrast, TACE alone was estimated to yield the lowest adjusted incremental cost ($1,494), followed by chemotherapy ($13,825), sorafenib alone ($19,706), and TACE+sorafenib ($24,420). Finally, TACE alone was found to be the most cost-effective strategy (ICER: $4,053/QALY, 95% CI -$30,000-$50,000/QALY) followed by TACE+sorafenib (ICER: $30,622/QALY, 95% CI $4,700-$71,000/QALY), chemotherapy (ICER: $47,911/QALY, 95% CI $13,000-$150,000/QALY), and sorafenib alone (ICER: $74,941/QALY, 95% CI $37,500-$204,000). See S8 Table, S9 Fig, S10 Fig and S11 Fig.

## Discussion

This study evaluated the real-world cost-effectiveness of non-curative palliative oncologic treatments such as TACE alone or TACE plus sorafenib, sorafenib alone, and non-sorafenib chemotherapy as compared to no treatment or BSC among patients diagnosed with HCC. Our results suggest that sorafenib treatment is the most widely used palliative treatment in advanced-stage HCC patients (68.8%), followed by chemotherapy (50%), and TACE alone or TACE plus sorafenib (37.7%). Compared with no treatment or BSC, the adjusted incremental benefit of TACE alone or TACE+sorafenib has been estimated to yield more units of LYs and QALYs and less cost than other non-curative palliative treatment options. The ICER of TACE alone or TACE+sorafenib treatment develops cost-effectiveness at a threshold of $6,665/QALY (95% CI: -$18,800-$34,500/QALY) which is below the commonly cited threshold per QALY of $50,000 [55]. The CEACs show that if a threshold of $50,000/QALY gained is to be chosen, TACE alone or TACE plus sorafenib would have a cost-effectiveness probability of 99.7%. Our cost-effectiveness results provide evidence that TACE alone or TACE plus sorafenib treatment appears to be acceptable treatment for patients with potential intermediate- or advanced-stage HCC in Ontario.

Non-curative palliative treatments are provided with a hope of providing HCC patients prolonged survival and improved quality of life. Unfortunately, advances have been modest. Although drug eluting beads theoretically could improve the efficacy and safety of TACE, a recent clinical and economic impact of drug eluting beads in TACE was unable to show improved prognosis in patients with unresectable HCC [56]. A recent meta-analysis evaluating the efficacy and safety of the combination therapy of TACE plus sorafenib in patients with intermediate- or advanced-stage of HCC suggests improved overall survival and time to progression, with tolerable toxicity compared to TACE alone [9]. Additionally, there is some evidence that TACE with adjuvant sorafenib is superior to sorafenib alone with respect to time to progression in patients with advanced-stage HCC [57]. A recent randomised controlled trial showed that regorafenib, an oral multikinase inhibitor, is the only systemic treatment shown to provide survival benefit in HCC patients progressing on first-line sorafenib treatment. This finding is associated with an increase in median survival from 7.8 months to 10.6 months [58].

HCC associated with chronic viral hepatitis has attendant increased rates of disease recurrence and poor survival. Control of hepatitic viral replication is an important prognostic intervention for infected patients, especially given recent advances in novel antiviral therapies [59]. Optimal outcomes in the cost effectiveness of HCC treatment necessitate patients at risk of HCC be diagnosed early and referred for treatment in a timely manner, leading to a better prognosis with multidisciplinary involvement.

The advantage of using net benefit framework in our study is that influential covariates can be adjusted for in the regression model to obtain a more accurate INB [35]. This NBR found several covariates associated with INB (*P* < 0.05), including age group and sex (from *λ* $10,000 to *λ* $100,000), Charlson-Deyo comorbidity index (from *λ* $0 to *λ* $100,000), and intermediate and advanced cancer stage (from *λ* $60,000 to *λ* $100,000).

A sensitivity analysis of multiple imputation for variables of missing data and adjusting for confounding covariates appeared robust to the base case relating to the incremental effects and costs and ICER of non-curative palliative treatments relative to no treatment or BSC. The effect and cost estimates through multiple imputation would be well suited to analyses of administrative claims data in which some covariates are missing.

There are a number of limitations in this study that should be noted. First, there is possibility of confounding by indication involving factors that are not observed, measured, or captured in routinely collected data in administrative health databases. Utilization of non-curative palliative treatment for HCC such as TACE, sorafenib, and chemotherapy seem low. The management of HCC is complex due to the underlying conditions which needs to be performed in a multidisciplinary approach [60,61]. Second, our analysis included a “sorafenib alone” sub-group based on the information available to us in our databases. While it is true that sorafenib is not publicly funded for those under 65 years of age, there is no recorded data for this sub-population within our databases and thus further investigation on this group would not be possible without significant speculation and assumption. The authors feel that our current analysis of patients receiving sorafenib alone, despite being limited by the aforementioned factors, remain generalizable to other populations globally as it provides a cost-effectiveness assessment of patients receiving sorafenib with large numbers in each subgroup. Third, our analysis considered only one course of each palliative monotherapy among patients receiving multiple courses. A recent study in North America found that Medicare expenditures doubled between receiving one and four or more TACE treatments, but expenses were distributed over more than an additional year of life [62]. Fourth, in our study, the majority of patients did not receive ultrasound screening (56.9%), especially those receiving sorafenib (62.4%), chemotherapy (54.6%), or BSC (57.5%). The lack of screening may have represented a missed opportunity for more curative treatment options. Lastly, this analysis was not able to include patients who may also have undergone combined or sequential treatment modalities (e.g. curative and non-curative palliative treatments) which are effective in improving the outcome of patients with HCC.

## Conclusion

In summary, our data shows that compared with no treatment or BSC, TACE alone or TACE and sorafenib combination treatment has the largest incremental benefit and is cost-effective if a threshold of $50,000/QALY gained is to be chosen, making it the preferred strategy for patients with intermediate- or advanced-stage HCC. Further research into new combination treatment strategies that afford the best tumor response and cost-effectiveness analysis of such new treatments are needed to dictate policy for this difficult to manage disease.

## Supporting information

S1 Fig Selection criteria for the study sample. (TIF)

S2 Fig Efficiency frontier: Plot of incremental quality-adjusted life years (QALYs) and costs of non-curative palliative treatments. i) transarterial chemoembolization (TACE) alone or TACE+sorafenib; ii) non-sorafenib chemotherapy alone; and iii) sorafenib alone relative to lowest cost scenario (no treatment or best supportive care [BSC]): Sensitivity analysis according to (A) lower bound (-25%) and (B) upper bound (+25%) of mean health state utilities of disease stage according to published literature and assumption. The dotted diagonal line represents the ceiling ratio. If an intervention lies above the line, it will not be acceptable on cost-effectiveness grounds.
(TIF)

S3 Fig Efficiency frontier: plot of incremental quality-adjusted life years (QALYs) and costs of non-curative palliative treatments. Sensitivity analyses using pooled mean health state utilities by liver disease stage, health state utilities for incurable HCC or after disease progression.
(TIF)

S4 Fig Estimates of incremental net benefit (i.e. incremental cost-effectiveness ratio, ICER) and its 95% confidence intervals as a function of willingness-to-pay threshold for an additional life year. Sensitivity analyses using pooled mean health state utilities by liver disease stage, health state utilities for incurable HCC or after disease progression.
(TIF)

S5 Fig Cost-effectiveness acceptability curves showing the probability that each non-curative palliative treatment strategy. i) TACE alone or TACE+sorafenib; ii) non-sorafenib chemotherapy alone; and iii) sorafenib alone relative to lowest cost scenario (no treatment or BSC). Sensitivity analyses using pooled mean health state utilities by liver disease stage, health state utilities for incurable HCC or after disease progression.
(TIF)

S6 Fig **Efficiency frontier: plot of incremental (A) life years (LYs) and (B) quality-adjusted life years (QALYs) and costs of non-curative palliative treatments:** i) TACE alone or TACE+sorafenib; ii) non-sorafenib chemotherapy alone; and iii) sorafenib alone relative to lowest cost scenario (no treatment or BSC): Sensitivity analysis according to multiple imputation for variables of missing data.
(TIF)

S7 Fig Estimates of incremental net benefit (i.e. incremental cost-effectiveness ratio, ICER) and its 95% confidence intervals as a function of willingness-to-pay threshold for an additional life year. (A) TACE alone or TACE+sorafenib vs. no treatment or BSC; (B) non-sorafenib chemotherapy alone vs. BSC; and (C) sorafenib alone vs. BSC; and for an additional QALY: (D) TACE alone or TACE+sorafenib vs. BSC; (E) non-sorafenib chemotherapy alone vs. BSC; and (F) sorafenib alone vs. BSC. Sensitivity analysis according to multiple imputation for variables of missing data.
(TIF)

S8 Fig Cost-effectiveness acceptability curves showing the probability that each non-curative palliative treatment strategy. i) TACE alone or TACE+sorafenib; ii) non-sorafenib chemotherapy alone; or iii) sorafenib alone is cost-effective compared with no treatment or BSC for a given willingness-to-pay threshold for an additional (A) life year (LY); and (B) quality adjusted life year (QALY). Sensitivity analysis according to multiple imputation for variables of missing data.
(TIF)

S9 Fig **Efficiency frontier: plot of incremental (A) life years (LYs) and (B) quality-adjusted life years (QALYs) and costs of non-curative palliative treatments:** i) TACE alone; ii) TACE+sorafenib; iii) non-sorafenib chemotherapy alone; and iv) sorafenib alone relative to lowest cost scenario (no treatment or BSC): Sensitivity analysis according to multiple imputation for variables of missing data.
(TIF)

S10 Fig Estimates of incremental net benefit (i.e. incremental cost-effectiveness ratio, ICER) and its 95% confidence intervals as a function of willingness-to-pay threshold for an additional QALY. (A) TACE alone vs. no treatment or BSC; (B) TACE+sorafenib vs. no treatment or BSC; (C) non-sorafenib chemotherapy alone vs. BSC; and (D) sorafenib alone vs. BSC. Sensitivity analysis according to multiple imputation for variables of missing data.
(TIF)

S11 Fig Cost-effectiveness acceptability curves showing the probability that each non-curative palliative treatment strategy. i) TACE alone; ii) TACE+sorafenib; iii) non-sorafenib chemotherapy alone; or iv) sorafenib alone is cost-effective compared with no treatment or BSC for a given willingness-to-pay threshold for an additional (A) life year (LY); and (B) quality adjusted life year (QALY). Sensitivity analysis according to multiple imputation for variables of missing data.
(TIF)

S1 Table Mean health state utilities of hepatocellular carcinoma by stage. (DOCX)

S2 Table Estimation of utilities for non-cirrhosis. (DOCX)

S3 Table Estimation of utilities for compensated cirrhosis. (DOCX)

S4 Table Estimation of utilities for decompensated cirrhosis. (DOCX)

S5 Table Estimation of utilities for hepatocellular carcinoma. (DOCX)

S6 Table Baseline characteristics of patients with hepatocellular carcinoma, 2007–2010. (DOCX)

S7 Table Estimates of incremental net benefit and probability of cost-effectiveness of non-curative palliative treatment strategies for hepatocellular carcinoma compared with no treatment or best supportive care as a function of willingness-to-pay threshold per additional life year over the study period 2007–2010. (DOCX)

S8 Table Adjusted incremental effects, incremental costs, and incremental cost-effectiveness ratios of non-curative palliative treatment strategies for hepatocellular carcinoma compared with no treatment or best supportive care, 2007–2010: net benefit regression, sensitivity analysis according to multiple imputation for variables of missing data. (DOCX)

## Abbreviations

HCC: hepatocellular carcinoma
TACE: transarterial chemoembolization
BSC: best supportive care
ICER: incremental cost-effectiveness ratio
LY: life year
QALY: quality-adjusted life year
INB: incremental net benefit
OCR: Ontario Cancer Registry
ICD-9: International Statistical Classification of Disease and Related Health Problems, 9th Revision
OHIP: Ontario Health Insurance Plan
ODB: Ontario Drug Benefit
ALD: alcoholic liver disease
PYLL: potential years of life lost
QALYL: quality-adjusted life years lost
CEAC: cost-effectiveness acceptability curve
CI: confidence interval
