# Supplementary figures and images for: Cost-effectiveness analysis of treatment with non-curative or palliative intent for hepatocellular carcinoma in the real-world setting
Source: PLoS One. 2017 Oct 10;12(10):e0185198. doi: 10.1371/journal.pone.0185198 (PMC5634563; doi:10.1371/journal.pone.0185198)

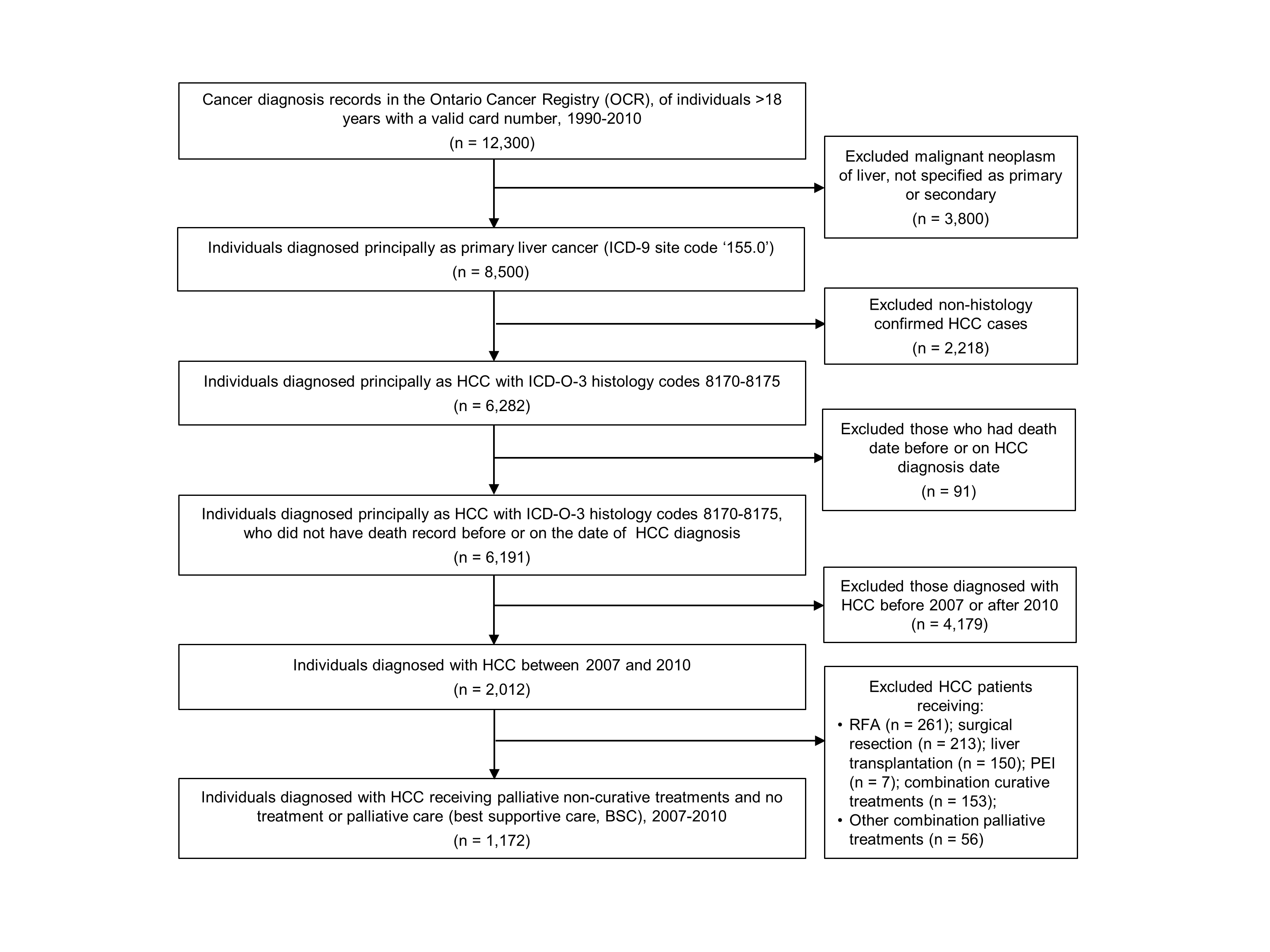

Supplement: S1 Fig — (TIF) [file pone.0185198.s001.tif]

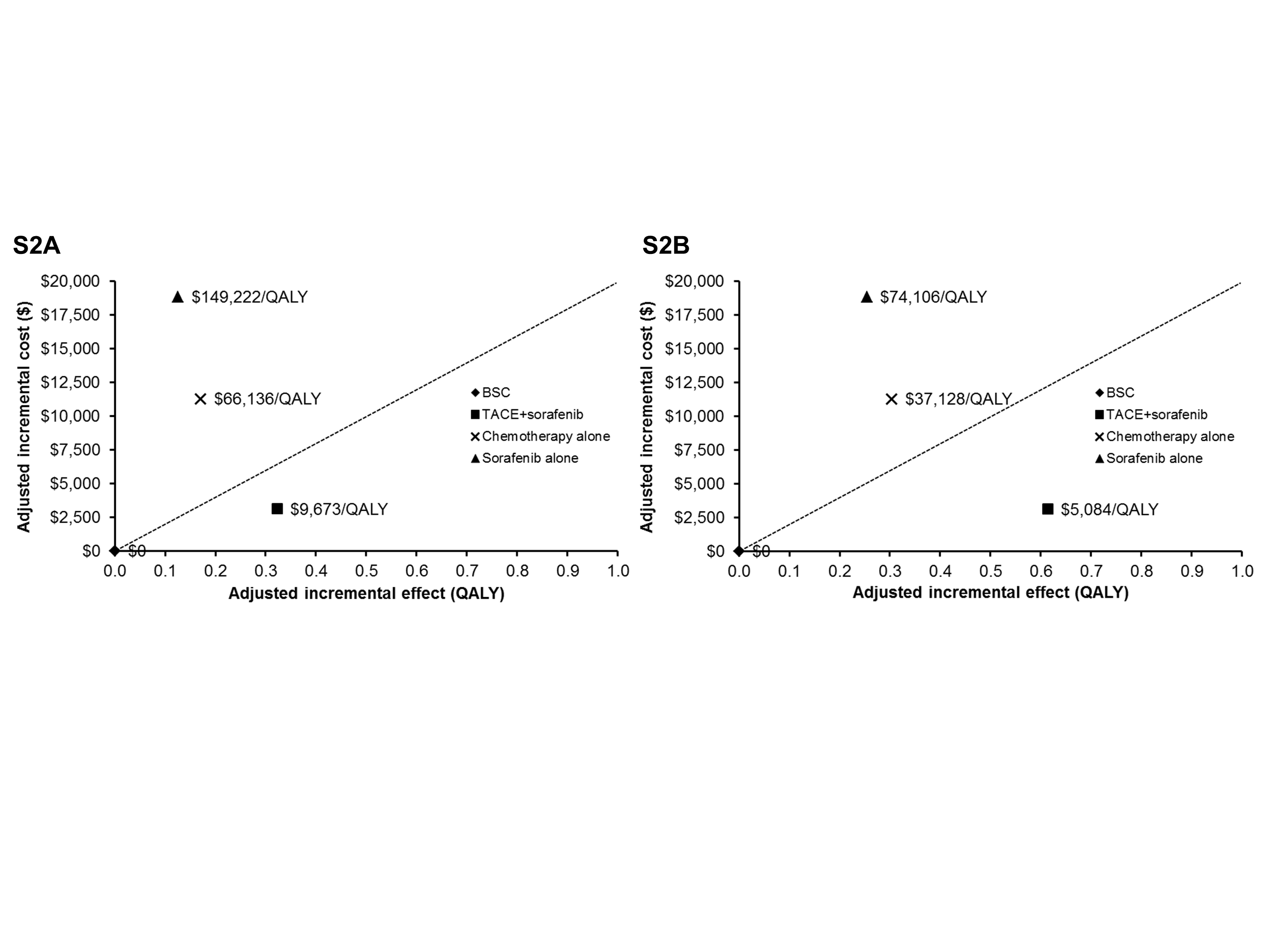

Supplement: S2 Fig — i) transarterial chemoembolization (TACE) alone or TACE+sorafenib; ii) non-sorafenib chemotherapy alone; and iii) sorafenib alone relative to lowest cost scenario (no treatment or best supportive care [BSC]): Sensitivity analysis according to (A) lower bound (-25%) and (B) upper bound (+25%) of mean health state utilities of disease stage according to published literature and assumption. The dotted diagonal line represents the ceiling ratio. If an intervention lies above the line, it will not be acceptable on cost-effectiveness grounds. (TIF) [file pone.0185198.s002.tif]

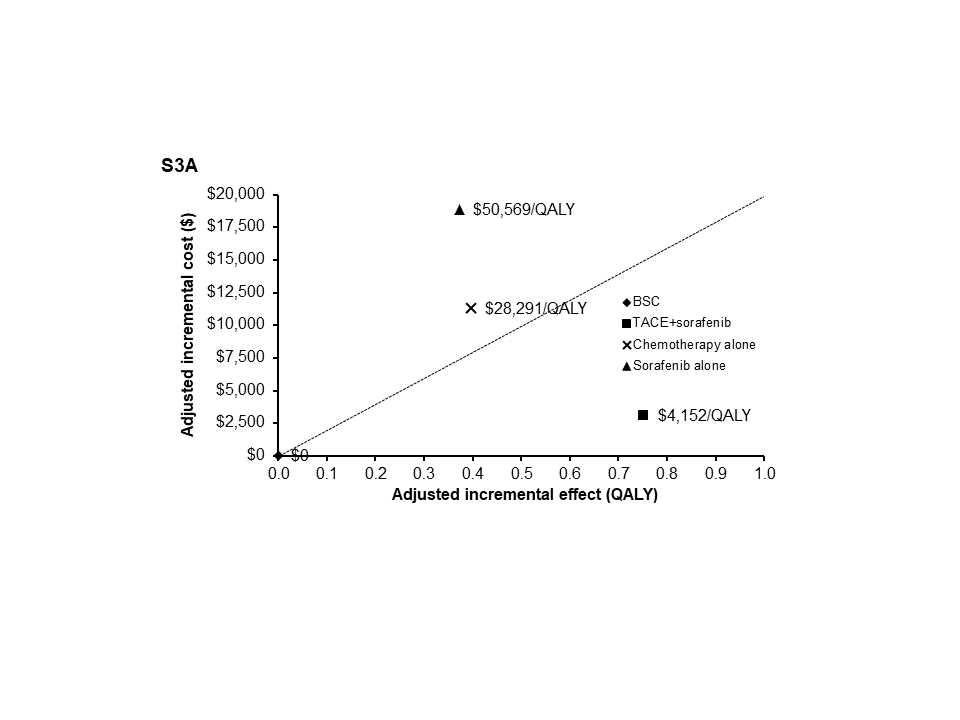

Supplement: S3 Fig — Sensitivity analyses using pooled mean health state utilities by liver disease stage, health state utilities for incurable HCC or after disease progression. (TIF) [file pone.0185198.s003.tif]

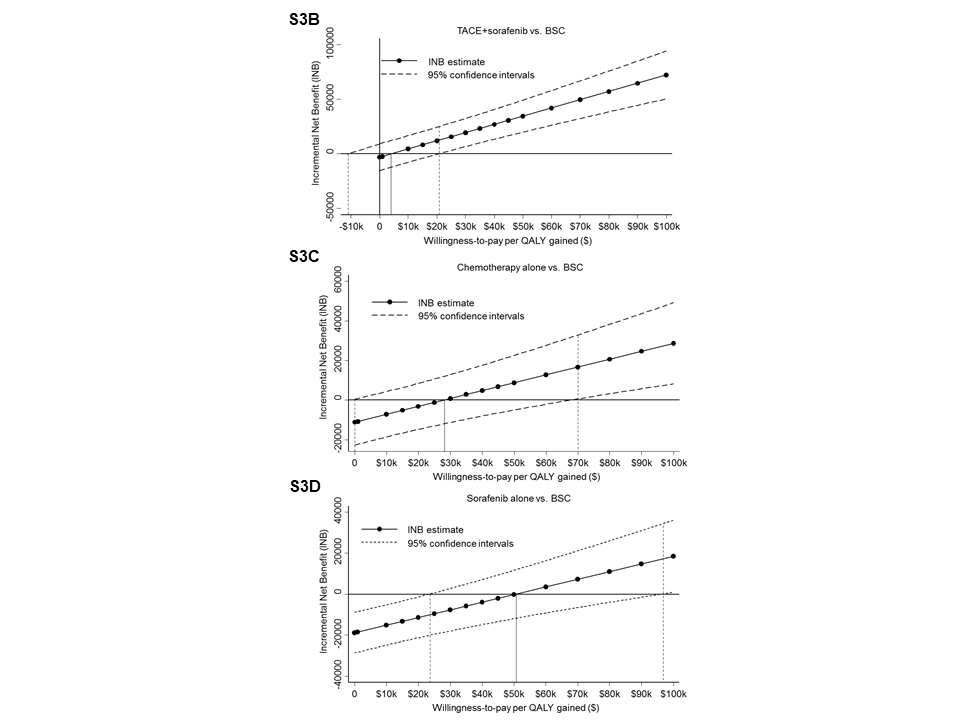

Supplement: S4 Fig — Sensitivity analyses using pooled mean health state utilities by liver disease stage, health state utilities for incurable HCC or after disease progression. (TIF) [file pone.0185198.s004.tif]

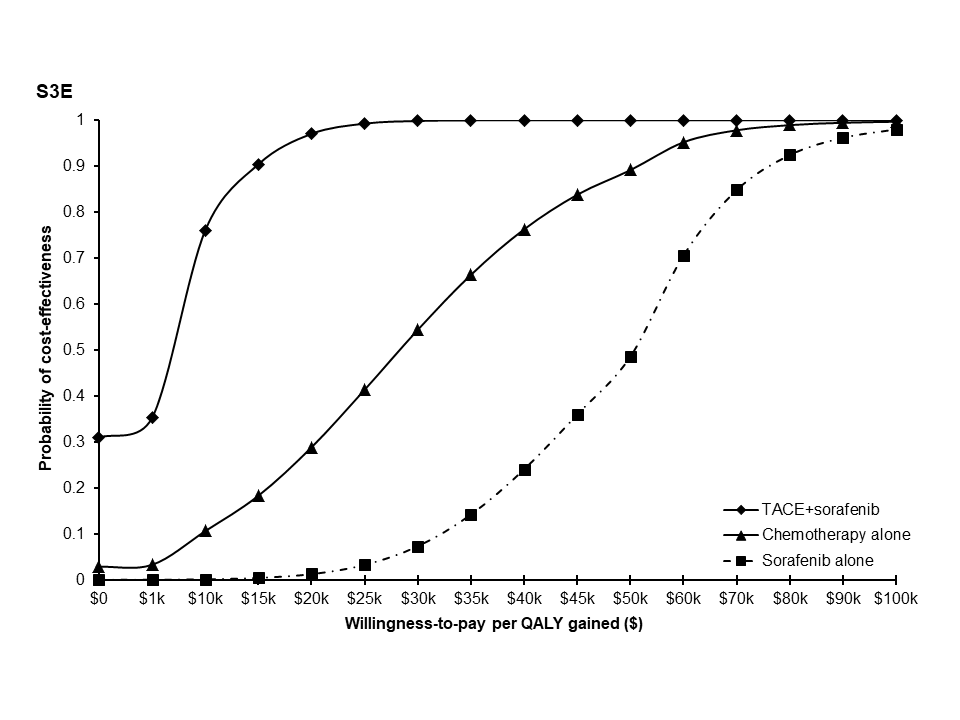

Supplement: S5 Fig — i) TACE alone or TACE+sorafenib; ii) non-sorafenib chemotherapy alone; and iii) sorafenib alone relative to lowest cost scenario (no treatment or BSC). Sensitivity analyses using pooled mean health state utilities by liver disease stage, health state utilities for incurable HCC or after disease progression. (TIF) [file pone.0185198.s005.tif]

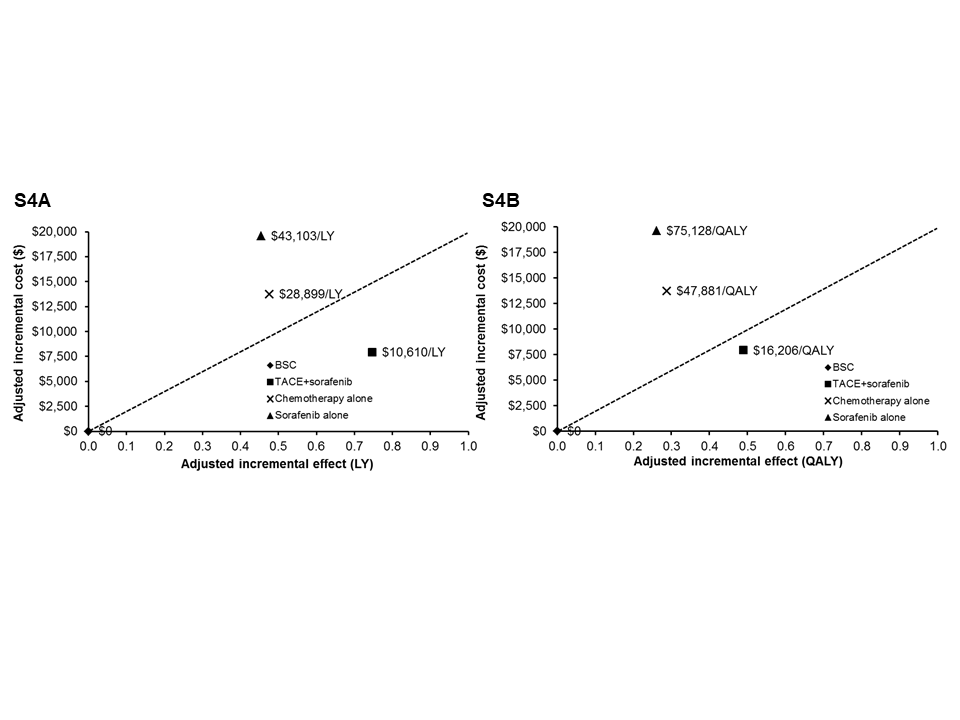

Supplement: S6 Fig — Efficiency frontier: plot of incremental (A) life years (LYs) and (B) quality-adjusted life years (QALYs) and costs of non-curative palliative treatments: i) TACE alone or TACE+sorafenib; ii) non-sorafenib chemotherapy alone; and iii) sorafenib alone relative to lowest cost scenario (no treatment or BSC): Sensitivity analysis according to multiple imputation for variables of missing data. (TIF) [file pone.0185198.s006.tif]

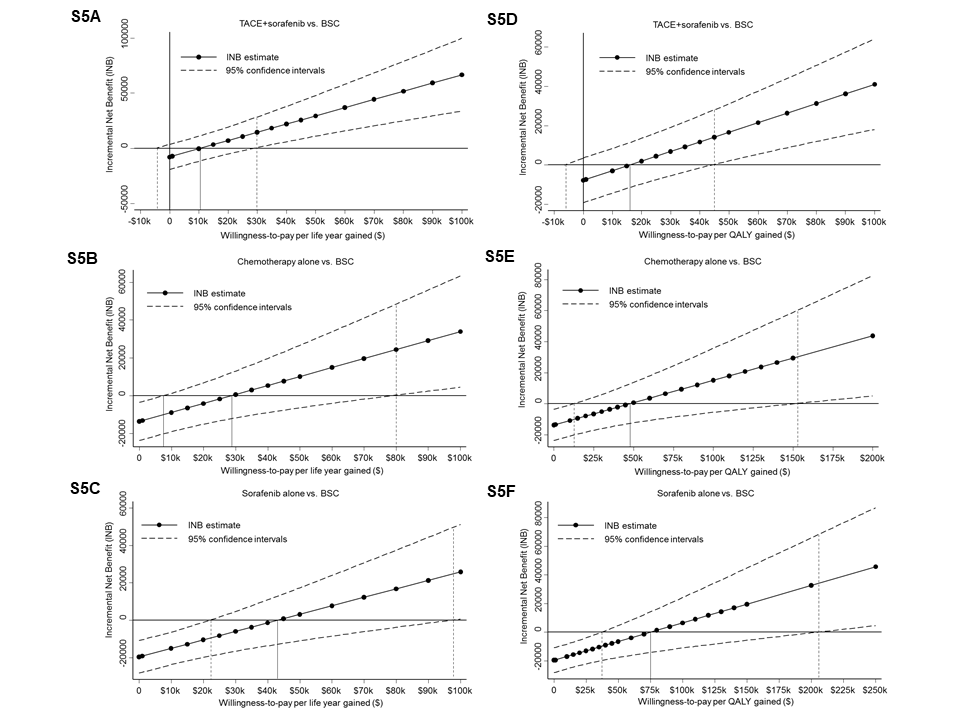

Supplement: S7 Fig — (A) TACE alone or TACE+sorafenib vs. no treatment or BSC; (B) non-sorafenib chemotherapy alone vs. BSC; and (C) sorafenib alone vs. BSC; and for an additional QALY: (D) TACE alone or TACE+sorafenib vs. BSC; (E) non-sorafenib chemotherapy alone vs. BSC; and (F) sorafenib alone vs. BSC. Sensitivity analysis according to multiple imputation for variables of missing data. (TIF) [file pone.0185198.s007.tif]

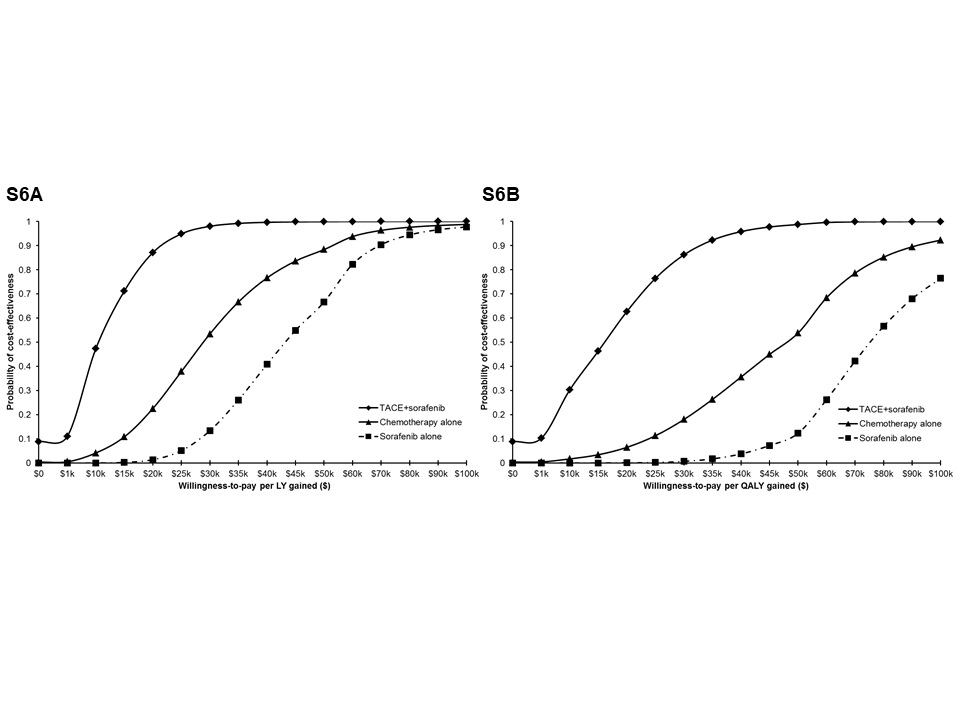

Supplement: S8 Fig — i) TACE alone or TACE+sorafenib; ii) non-sorafenib chemotherapy alone; or iii) sorafenib alone is cost-effective compared with no treatment or BSC for a given willingness-to-pay threshold for an additional (A) life year (LY); and (B) quality adjusted life year (QALY). Sensitivity analysis according to multiple imputation for variables of missing data. (TIF) [file pone.0185198.s008.tif]

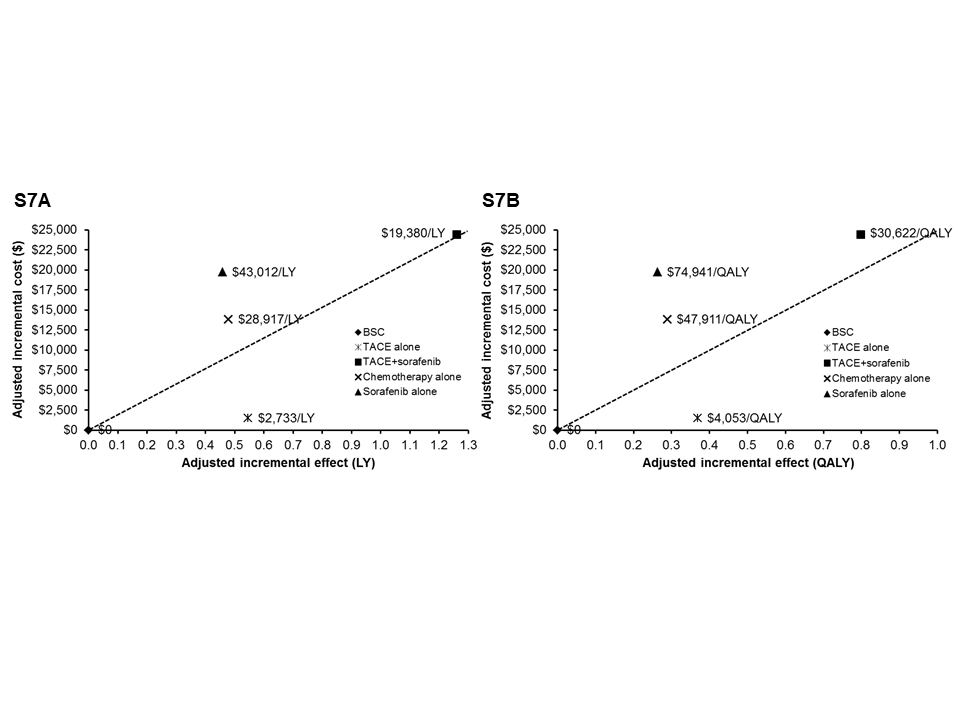

Supplement: S9 Fig — Efficiency frontier: plot of incremental (A) life years (LYs) and (B) quality-adjusted life years (QALYs) and costs of non-curative palliative treatments: i) TACE alone; ii) TACE+sorafenib; iii) non-sorafenib chemotherapy alone; and iv) sorafenib alone relative to lowest cost scenario (no treatment or BSC): Sensitivity analysis according to multiple imputation for variables of missing data. (TIF) [file pone.0185198.s009.tif]

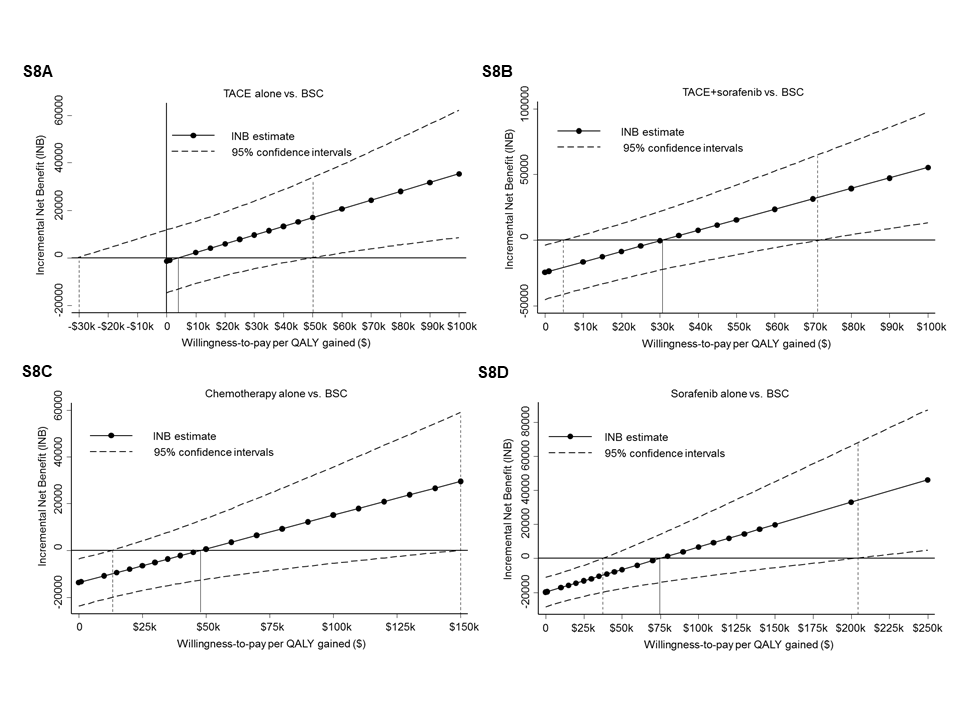

Supplement: S10 Fig — (A) TACE alone vs. no treatment or BSC; (B) TACE+sorafenib vs. no treatment or BSC; (C) non-sorafenib chemotherapy alone vs. BSC; and (D) sorafenib alone vs. BSC. Sensitivity analysis according to multiple imputation for variables of missing data. (TIF) [file pone.0185198.s010.tif]

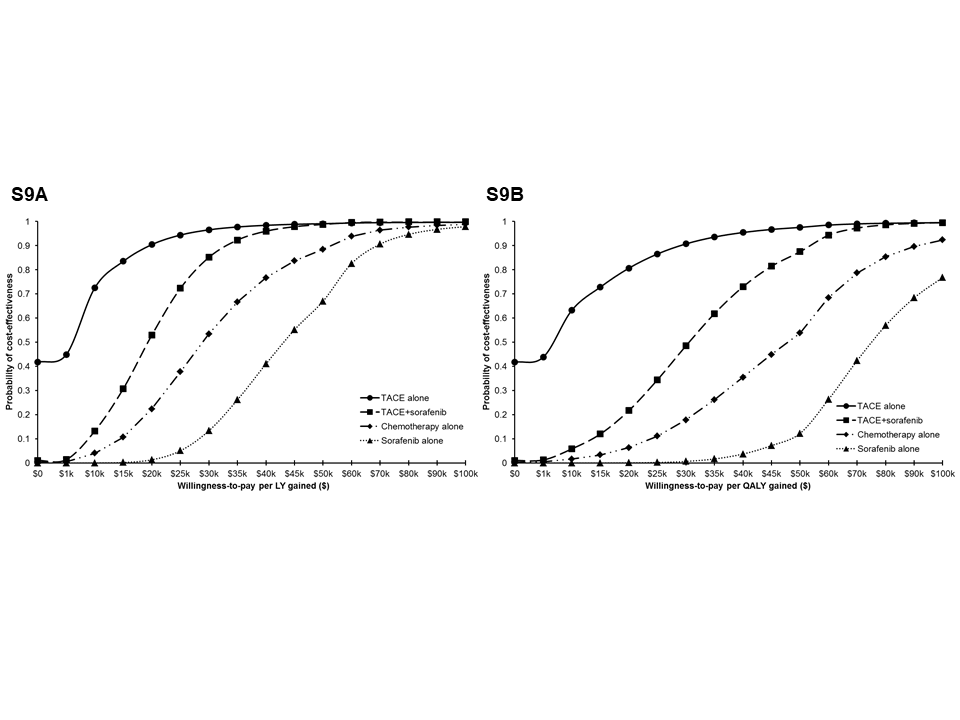

Supplement: S11 Fig — i) TACE alone; ii) TACE+sorafenib; iii) non-sorafenib chemotherapy alone; or iv) sorafenib alone is cost-effective compared with no treatment or BSC for a given willingness-to-pay threshold for an additional (A) life year (LY); and (B) quality adjusted life year (QALY). Sensitivity analysis according to multiple imputation for variables of missing data. (TIF) [file pone.0185198.s011.tif]
