# Supplementary material for: Cost-effectiveness analysis of treatment with non-curative or palliative intent for hepatocellular carcinoma in the real-world setting
Source: PLoS One. 2017 Oct 10;12(10):e0185198. doi: 10.1371/journal.pone.0185198 (PMC5634563; doi:10.1371/journal.pone.0185198)
Supplement: S1 Table — (DOCX) [file pone.0185198.s012.docx]

**S1 Table. Mean health state utilities of hepatocellular carcinoma by stage**

| Stage at diagnosis |  |  | Range^*^ | | Reference / Assumption |
| --- | --- | --- | --- | --- | --- |
|  | n | Mean | Lower Bound | Upper Bound |  |
| Early (stage I) | 67 | 0.7785 | 0.5839 | 0.9731 | Assumption |
| Intermediate (stage II) | 106 | 0.7200 | 0.5400 | 0.9000 | [[1-3](#_ENREF_1)] |
| Advanced (stage III) | 334 | 0.6615 | 0.4961 | 0.8269 | Assumption |
| Terminal (stage IV) | 155 | 0.6030 | 0.4523 | 0.7538 | Assumption |

^*^+25% sensitivity analysis.

**References**

1. Hsu PC, Federico CA, Krajden M, Yoshida EM, Bremner KE, Anderson FH, et al. Health utilities and psychometric quality of life in patients with early- and late-stage hepatitis C virus infection. J Gastroenterol Hepatol. 2012; 27:149-157.

2. Chong CA, Gulamhussein A, Heathcote EJ, Lilly L, Sherman M, Naglie G, et al. Health-state utilities and quality of life in hepatitis C patients. Am J Gastroenterol. 2003; 98:630-638.

3. Thein HH, Krahn M, Kaldor JM, Dore GJ. Estimation of utilities for chronic hepatitis C from SF-36 scores. Am J Gastroenterol. 2005; 100:643-651.
