# Supplementary material for: Cost-effectiveness analysis of treatment with non-curative or palliative intent for hepatocellular carcinoma in the real-world setting
Source: PLoS One. 2017 Oct 10;12(10):e0185198. doi: 10.1371/journal.pone.0185198 (PMC5634563; doi:10.1371/journal.pone.0185198)
Supplement: S3 Table — (DOCX) [file pone.0185198.s014.docx]

**S3 Table. Estimation of utilities for compensated cirrhosis**

| Author, year | Preference-based measures | Country | Disease | Mean | Standard error | Lower limit | Upper limit |
| --- | --- | --- | --- | --- | --- | --- | --- |
| Chong et al, 2003[[1](#_ENREF_1)] | EQ-5D | Canada | HCV | 0.740 | 0.050 | 0.642 | 0.838 |
| Chong et al, 2003[[1](#_ENREF_1)] | HUI3 | Canada | HCV | 0.740 | 0.050 | 0.642 | 0.838 |
| Chong et al, 2003[[1](#_ENREF_1)] | SG | Canada | HCV | 0.800 | 0.050 | 0.702 | 0.898 |
| Sherman et al, 2004[[2](#_ENREF_2)] | SG | US | HCV | 0.830 | 0.040 | 0.752 | 0.908 |
| Sherman et al, 2004[[2](#_ENREF_2)] | TTO | US | HCV | 0.900 | 0.030 | 0.841 | 0.959 |
| Siebert et al, 2001[[3](#_ENREF_3), [4](#_ENREF_4)] | EQ-5D | Germany | HCV | 0.740 | 0.020 | 0.701 | 0.779 |
| Younossi et al, 2001[[5](#_ENREF_5)] | HUI2 | US | HCV | 0.820 | 0.040 | 0.742 | 0.898 |
| Hsu et al, 2012[[6](#_ENREF_6)] | HUI2 | Canada | HCV | 0.730 | 0.012 | 0.706 | 0.754 |
| Hsu et al, 2012[[6](#_ENREF_6)] | SF-6D | Canada | HCV | 0.660 | 0.008 | 0.644 | 0.676 |
| Hsu et al, 2012[[6](#_ENREF_6)] | TTO | Canada | HCV | 0.780 | 0.021 | 0.739 | 0.821 |
| Levy et al. 2008[[7](#_ENREF_7)] | SG | US, Canada, UK, Spain, China, HK | HBV | 0.690 | 0.013 | 0.665 | 0.715 |
| Woo et al. 2012[[8](#_ENREF_8)] | EQ-5D | Canada | HBV | 0.880 | 0.018 | 0.845 | 0.915 |
| Woo et al. 2012[[8](#_ENREF_8)] | HUI3 | Canada | HBV | 0.810 | 0.028 | 0.755 | 0.865 |
| Woo et al. 2012[[8](#_ENREF_8)] | SG | Canada | HBV | 0.870 | 0.020 | 0.830 | 0.910 |
| Fixed |  |  |  | 0.731 | 0.005 | 0.722 | 0.741 |
| Random |  |  |  | 0.784 | 0.024 | 0.737 | 0.831 |

Assessment of heterogeneity: *I*^2^ = 95.1%; *P* < 0.001.

**References**

1. Chong CA, Gulamhussein A, Heathcote EJ, Lilly L, Sherman M, Naglie G, et al. Health-state utilities and quality of life in hepatitis C patients. Am J Gastroenterol. 2003; 98:630-638.

2. Sherman KE, Sherman SN, Chenier T, Tsevat J. Health values of patients with chronic hepatitis C infection. Arch Intern Med. 2004; 164:2377-2382.

3. Siebert U, Sieberer R, Greiner W, et al. Patient-based health-related quality of life in different stages of chronic hepatitis C [Abstract]. Hepatology 2001;34(Pt 2): AB222A.

4. McLernon DJ, Dillon J, Donnan PT. Health-state utilities in liver disease: a systematic review. Med Decis Making. 2008; 28:582-592.

5. Younossi ZM, Boparai N, McCormick M, Price LL, Guyatt G. Assessment of utilities and health-related quality of life in patients with chronic liver disease. Am J Gastroenterol. 2001; 96:579-583.

6. Hsu PC, Federico CA, Krajden M, Yoshida EM, Bremner KE, Anderson FH, et al. Health utilities and psychometric quality of life in patients with early- and late-stage hepatitis C virus infection. J Gastroenterol Hepatol. 2012; 27:149-157.

7. Levy AR, Kowdley KV, Iloeje U, Tafesse E, Mukherjee J, Gish R, et al. The impact of chronic hepatitis B on quality of life: a multinational study of utilities from infected and uninfected persons. Value Health. 2008; 11:527-538.

8. Woo G, Tomlinson G, Yim C, Lilly L, Therapondos G, Wong DK, et al. Health state utilities and quality of life in patients with hepatitis B. Can J Gastroenterol. 2012; 26:445-451.
