# Supplementary material for: Cost-effectiveness analysis of treatment with non-curative or palliative intent for hepatocellular carcinoma in the real-world setting
Source: PLoS One. 2017 Oct 10;12(10):e0185198. doi: 10.1371/journal.pone.0185198 (PMC5634563; doi:10.1371/journal.pone.0185198)
Supplement: S4 Table — (DOCX) [file pone.0185198.s015.docx]

**S4 Table. Estimation of utilities for decompensated cirrhosis**

| Author, year | Preference-based measures | Country | Disease | Mean | Standard error | Lower limit | Upper limit |
| --- | --- | --- | --- | --- | --- | --- | --- |
| Chong et al, 2003[[1](#_ENREF_1)] | EQ-5D | Canada | HCV | 0.660 | 0.100 | 0.464 | 0.856 |
| Chong et al, 2003[[1](#_ENREF_1)] | HUI3 | Canada | HCV | 0.690 | 0.080 | 0.533 | 0.847 |
| Chong et al, 2003[[1](#_ENREF_1)] | SG | Canada | HCV | 0.600 | 0.120 | 0.365 | 0.835 |
| Sherman et al, 2004[[2](#_ENREF_2)] | SG | US | HCV | 0.720 | 0.120 | 0.485 | 0.955 |
| Sherman et al, 2004[[2](#_ENREF_2)] | TTO | US | HCV | 0.720 | 0.120 | 0.485 | 0.955 |
| Siebert et al, 2001[[3](#_ENREF_3), [4](#_ENREF_4)] | EQ-5D | Germany | HCV | 0.720 | 0.030 | 0.661 | 0.779 |
| Younossi et al, 2001[[5](#_ENREF_5)] | HUI2 | US | HCV | 0.710 | 0.100 | 0.514 | 0.906 |
| Levy et al. 2008[[6](#_ENREF_6)] | SG | US, Canada, UK, Spain, China, HK | HBV | 0.350 | 0.013 | 0.325 | 0.375 |
| Woo et al. 2012[[7](#_ENREF_7)] | EQ-5D | Canada | HBV | 0.730 | 0.156 | 0.425 | 1.035 |
| Woo et al. 2012[[7](#_ENREF_7)] | HUI3 | Canada | HBV | 0.490 | 0.135 | 0.225 | 0.755 |
| Woo et al. 2012[[7](#_ENREF_7)] | SG | Canada | HBV | 0.820 | 0.102 | 0.620 | 1.020 |
| Fixed |  |  |  | 0.434 | 0.011 | 0.412 | 0.456 |
| Random |  |  |  | 0.652 | 0.078 | 0.498 | 0.805 |

Assessment of heterogeneity: *I*^2^ = 94.7%; *P* < 0.001.

**References**

1. Chong CA, Gulamhussein A, Heathcote EJ, Lilly L, Sherman M, Naglie G, et al. Health-state utilities and quality of life in hepatitis C patients. Am J Gastroenterol. 2003; 98:630-638.

2. Sherman KE, Sherman SN, Chenier T, Tsevat J. Health values of patients with chronic hepatitis C infection. Arch Intern Med. 2004; 164:2377-2382.

3. Siebert U, Sieberer R, Greiner W, et al. Patient-based health-related quality of life in different stages of chronic hepatitis C [Abstract]. Hepatology 2001;34(Pt 2): AB222A.

4. McLernon DJ, Dillon J, Donnan PT. Health-state utilities in liver disease: a systematic review. Med Decis Making. 2008; 28:582-592.

5. Younossi ZM, Boparai N, McCormick M, Price LL, Guyatt G. Assessment of utilities and health-related quality of life in patients with chronic liver disease. Am J Gastroenterol. 2001; 96:579-583.

6. Levy AR, Kowdley KV, Iloeje U, Tafesse E, Mukherjee J, Gish R, et al. The impact of chronic hepatitis B on quality of life: a multinational study of utilities from infected and uninfected persons. Value Health. 2008; 11:527-538.

7. Woo G, Tomlinson G, Yim C, Lilly L, Therapondos G, Wong DK, et al. Health state utilities and quality of life in patients with hepatitis B. Can J Gastroenterol. 2012; 26:445-451.
