# Supplementary material for: Cost-effectiveness analysis of treatment with non-curative or palliative intent for hepatocellular carcinoma in the real-world setting
Source: PLoS One. 2017 Oct 10;12(10):e0185198. doi: 10.1371/journal.pone.0185198 (PMC5634563; doi:10.1371/journal.pone.0185198)
Supplement: S5 Table — (DOCX) [file pone.0185198.s016.docx]

**S5 Table. Estimation of utilities for hepatocellular carcinoma**

| Author, year | Preference-based measures | Country | Disease | Mean | Standard error | Lower limit | Upper limit |
| --- | --- | --- | --- | --- | --- | --- | --- |
| Chong et al, 2003[[1](#_ENREF_1)] | EQ-5D | Canada | HCV | 0.650 | 0.107 | 0.440 | 0.860 |
| Chong et al, 2003[[1](#_ENREF_1)] | HUI3 | Canada | HCV | 0.510 | 0.127 | 0.261 | 0.759 |
| Chong et al, 2003[[1](#_ENREF_1)] | SG | Canada | HCV | 0.720 | 0.051 | 0.620 | 0.820 |
| Hsu et al, 2012[[2](#_ENREF_2)] | HUI2 | Canada | HCV | 0.720 | 0.056 | 0.610 | 0.830 |
| Hsu et al, 2012[[2](#_ENREF_2)] | SF-6D | Canada | HCV | 0.610 | 0.027 | 0.557 | 0.663 |
| Hsu et al, 2012[[2](#_ENREF_2)] | TTO | Canada | HCV | 0.780 | 0.054 | 0.674 | 0.886 |
| Levy et al. 2008[[3](#_ENREF_3)] | SG | US, Canada, UK, Spain, China, HK | HBV | 0.380 | 0.013 | 0.355 | 0.405 |
| Woo et al. 2012[[4](#_ENREF_4)] | EQ-5D | Canada | HBV | 0.810 | 0.069 | 0.675 | 0.945 |
| Woo et al. 2012[[4](#_ENREF_4)] | HUI3 | Canada | HBV | 0.850 | 0.049 | 0.755 | 0.945 |
| Woo et al. 2012[[4](#_ENREF_4)] | SG | Canada | HBV | 0.840 | 0.038 | 0.765 | 0.915 |
| Fixed |  |  |  | 0.513 | 0.010 | 0.493 | 0.533 |
| Random |  |  |  | 0.689 | 0.075 | 0.543 | 0.836 |

Assessment of heterogeneity: *I*^2^ = 97.2%; *P* < 0.001.

Incurable hepatocellular carcinoma utility: mean 0.40; plausible range 0.32-0.48 [[5](#_ENREF_5)].

**References**

1. Chong CA, Gulamhussein A, Heathcote EJ, Lilly L, Sherman M, Naglie G, et al. Health-state utilities and quality of life in hepatitis C patients. Am J Gastroenterol. 2003; 98:630-638.

2. Hsu PC, Federico CA, Krajden M, Yoshida EM, Bremner KE, Anderson FH, et al. Health utilities and psychometric quality of life in patients with early- and late-stage hepatitis C virus infection. J Gastroenterol Hepatol. 2012; 27:149-157.

3. Levy AR, Kowdley KV, Iloeje U, Tafesse E, Mukherjee J, Gish R, et al. The impact of chronic hepatitis B on quality of life: a multinational study of utilities from infected and uninfected persons. Value Health. 2008; 11:527-538.

4. Woo G, Tomlinson G, Yim C, Lilly L, Therapondos G, Wong DK, et al. Health state utilities and quality of life in patients with hepatitis B. Can J Gastroenterol. 2012; 26:445-451.

5. Cucchetti A, Piscaglia F, Cescon M, Colecchia A, Ercolani G, Bolondi L, et al. Cost-effectiveness of hepatic resection versus percutaneous radiofrequency ablation for early hepatocellular carcinoma. J Hepatol. 2013; 59:300-307.
