# Supplementary material for: Cost-effectiveness analysis of treatment with non-curative or palliative intent for hepatocellular carcinoma in the real-world setting
Source: PLoS One. 2017 Oct 10;12(10):e0185198. doi: 10.1371/journal.pone.0185198 (PMC5634563; doi:10.1371/journal.pone.0185198)
Supplement: S6 Table — (DOCX) [file pone.0185198.s017.docx]

**S6 Table. Baseline characteristics of patients with hepatocellular carcinoma, 2007-2010**

| Variable | N (%) |
| --- | --- |
| Total | 1172 |
| Age group (years) |  |
| <60 | 323 (27.6) |
| 60-69 | 291 (24.8) |
| 70-79 | 348 (29.7) |
| 80+ | 210 (17.9) |
| Sex |  |
| Female | 248 (21.2) |
| Male | 924 (78.8) |
| Income quintile |  |
| Q1 (lowest) | 318 (27.1) |
| Q2 | 274 (23.4) |
| Q3 | 183 (15.6) |
| Q4 | 188 (16.0) |
| Q5 (highest) | 203 (17.3) |
| Missing | 6 (0.5) |
| Residence |  |
| Urban | 1047 (89.3) |
| Rural | 124 (10.6) |
| Missing | - |
| Birth country |  |
| Canada | 564 (48.1) |
| Other | 510 (43.5) |
| Unknown/Missing | 98 (8.4) |
| Charlson-Deyo comorbidity index |  |
| 0 | 401 (34.2) |
| 1 | 304 (25.9) |
| 2 | 130 (11.1) |
| 3+ | 76 (6.5) |
| No hospitalization record | 261 (22.3) |
| Diabetes diagnosis | 573 (48.9) |
| HIV | 18 (1.5) |

S6 Table continued on the following page

**S6 Table. Baseline characteristics of patients with hepatocellular carcinoma, 2007-2010 (continued)**

| **Variable** | **N (%)** |
| --- | --- |
| Indicators of liver disease stage |  |
| Viral hepatitis | 35 (3.0) |
| No cirrhosis | 312 (26.6) |
| Cirrhosis | 195 (16.6) |
| ALD + Cirrhosis | 35 (3.0) |
| Viral hepatitis + Cirrhosis | 38 (3.2) |
| Decompensated cirrhosis | 298 (25.4) |
| ALD + Decompensated cirrhosis | 152 (13) |
| Viral hepatitis + Decompensated cirrhosis | 38 (3.2) |
| ALD + Viral Hepatitis + Decompensated cirrhosis | 29 (2.5) |
| Ultrasound screening 2 years before HCC diagnosis |  |
| ≥1 screens annually | 80 (6.8) |
| Inconsistent screening | 425 (36.3) |
| No screening | 667 (56.9) |
| Stage at HCC diagnosis |  |
| Early (stage I) | 67 (5.7) |
| Intermediate (stage II) | 106 (9.0) |
| Advanced (stage III) | 334 (28.5) |
| Advanced (stage IV) | 155 (13.2) |
| Unknown/Missing | 510 (43.5) |
| Year of HCC diagnosis |  |
| 2007 | 283 (24.2) |
| 2008 | 257 (21.9) |
| 2009 | 304 (25.9) |
| 2010 | 328 (28.0) |

n = 1,172. ‘‘-“, counts less than six have been suppressed.

ALD, alcoholic liver disease; HCC, hepatocellular carcinoma.
