# Supplementary material for: Cost-effectiveness analysis of treatment with non-curative or palliative intent for hepatocellular carcinoma in the real-world setting
Source: PLoS One. 2017 Oct 10;12(10):e0185198. doi: 10.1371/journal.pone.0185198 (PMC5634563; doi:10.1371/journal.pone.0185198)
Supplement: S7 Table — (DOCX) [file pone.0185198.s018.docx]

**S7 Table. Estimates of incremental net benefit and probability of cost-effectiveness of non-curative palliative treatment strategies for hepatocellular carcinoma compared with no treatment or best supportive care as a function of willingness-to-pay threshold per additional life year over the study period 2007-2010**

| λ thresholds | TACE alone or TACE + Sorafenib | | |  | Sorafenib alone | | |  | Non-sorafenib chemotherapy alone | | |
| --- | --- | --- | --- | --- | --- | --- | --- | --- | --- | --- | --- |
|  | INB estimate (SE) | *P*-value^*^ | Probability of cost-effectiveness |  | INB estimate (SE) | *P*-value^*^ | Probability of cost-effectiveness |  | INB estimate (SE) | *P*-value^*^ | Probability of cost-effectiveness |
| $0 | -3120 (6284) | 0.310 | 31.0% |  | -18821 (5049) | <0.001 | 0% |  | -11263 (5898) | 0.028 | 2.8% |
| $1,000 | -2437 (6257) | 0.349 | 34.9% |  | -18506 (5027) | <0.001 | 0% |  | -10902 (5873) | 0.032 | 3.2% |
| $10,000 | 3708 (6175) | 0.274 | 72.6% |  | -15674 (4961) | 0.001 | 0.1% |  | -7649 (5796) | 0.094 | 9.4% |
| $20,000 | 10536 (6427) | 0.051 | 94.9% |  | -12526 (5163) | 0.008 | 0.8% |  | -4036 (6032) | 0.252 | 25.2% |
| $30,000 | 17365 (7000) | 0.007 | 99.3% |  | -9379 (5624) | 0.048 | 4.8% |  | -422 (6571) | 0.474 | 47.4% |
| $40,000 | 24193 (7826) | 0.001 | 99.9% |  | -6232 (6287) | 0.161 | 16.1% |  | 3192 (7346) | 0.332 | 66.8% |
| $50,000 | 31021 (8832) | <0.001 | 100% |  | -3084 (7096) | 0.332 | 33.2% |  | 6806 (8291) | 0.206 | 79.4% |
| $60,000 | 37850 (9965) | <0.001 | 100% |  | 63 (8007) | 0.497 | 50.3% |  | 10419 (9354) | 0.133 | 86.7% |
| $70,000 | 44678 (11187) | <0.001 | 100% |  | 3211 (8988) | 0.361 | 64.0% |  | 14033 (10501) | 0.091 | 90.9% |
| $80,000 | 51506 (12470) | <0.001 | 100% |  | 6358 (10019) | 0.263 | 73.7% |  | 17647 (11706) | 0.066 | 93.4% |
| $90,000 | 58335 (13799) | <0.001 | 100% |  | 9505 (11086) | 0.196 | 80.4% |  | 21260 (12952) | 0.051 | 94.9% |
| $100,000 | 65163 (15160) | <0.001 | 100% |  | 12653 (12180) | 0.150 | 85.0% |  | 24874 (14231) | 0.041 | 96.0% |

^*^one-sided *P*-value. λ, willingness-to-pay; INB, incremental net benefit; SE, standard error.
