# Supplementary material for: Cost-effectiveness analysis of treatment with non-curative or palliative intent for hepatocellular carcinoma in the real-world setting
Source: PLoS One. 2017 Oct 10;12(10):e0185198. doi: 10.1371/journal.pone.0185198 (PMC5634563; doi:10.1371/journal.pone.0185198)
Supplement: S8 Table — (DOCX) [file pone.0185198.s019.docx]

**S8 Table. Adjusted incremental effects, incremental costs, and incremental cost-effectiveness ratios of non-curative palliative treatment strategies for hepatocellular carcinoma compared with no treatment or best supportive care, 2007-2010: net benefit regression, sensitivity analysis according to multiple imputation for variables of missing data**

| Treatment Strategies | Average Total Effect (PYLL) | Average Total Effect (QALYL) | Average Total Cost ($) | Adjusted Incremental Effect^*^ (LYs) | Adjusted Incremental Effect^*^ (QALYs) | Adjusted Incremental Cost ($)^†^ | Adjusted ICER ($/LY gained) | Adjusted ICER ($/QALY gained) |
| --- | --- | --- | --- | --- | --- | --- | --- | --- |
| No treatment or BSC (n = 4800) | 11.5710 | 10.6226 | $36,415 |  |  |  |  |  |
| TACE alone (n = 190) | 11.5442 | 10.7188 | $40,241 | 0.54652 | 0.36852 | $1,494 | $2,733 | $4,053 |
| Non-sorafenib chemotherapy alone (n = 330) | 12.4255 | 11.5722 | $51,657 | 0.47809 | 0.28855 | $13,825 | $28,917 | $47,911 |
| Sorafenib alone (n = 465) | 10.4988 | 9.7664 | $53,198 | 0.45814 | 0.26295 | $19,706 | $43,012 | $74,941 |
| TACE + Sorafenib (n = 75) | 10.7860 | 10.0879 | $59,310 | 1.26008 | 0.79747 | $24,420 | $19,380 | $30,622 |

^*^Incremental effect is calculated as treatment effect minus no treatment or BSC effect, adjusted for relevant covariates (dummy variables), including age, sex, income quintile, urban/rural residence, birth country, Charlson-Deyo comorbidity index, diabetes, HIV, indicators of liver disease stage, ultrasound screening, stage at HCC diagnosis, and year of HCC diagnosis. Positive value indicates increase in the effect relative to “no treatment or BSC”.

^†^Incremental cost is calculated as treatment cost minus no treatment or BSC cost, adjusted for aforementioned covariates. Positive value indicates increase in cost relative to “no treatment or BSC”. Values are expressed as the mean. All costs reflect 2013 US$ per person.

BSC, best supportive care (formal palliative care); TACE, transarterial chemoembolization; PYLL, potential years of life lost; QALYL, quality-adjusted life years lost; LY, life year; QALY, quality-adjusted life years.
